# PP2Acα-B′/PR61 Holoenzyme of Toxoplasma gondii Is Required for the Amylopectin Metabolism and Proliferation of Tachyzoites

**DOI:** 10.1128/spectrum.00104-23

**Published:** 2023-05-18

**Authors:** Mingxiu Zhao, Yi Yang, Yue Shi, Xueqiu Chen, Yimin Yang, Lingtao Pan, Zhendong Du, Hongchao Sun, Chaoqun Yao, Guangxu Ma, Aifang Du

**Affiliations:** a Institute of Preventive Veterinary Medicine, Zhejiang Provincial Key Laboratory of Preventive Veterinary Medicine, College of Animal Sciences, Zhejiang University, Hangzhou, Zhejiang Province, China; b Hainan Institute, Zhejiang University, Yazhou Bay Sci-Tech City, Sanya, China; c Department of Animal Parasitology, Institute of Animal Husbandry and Veterinary Medicine, Zhejiang Academy of Agricultural Science, Hangzhou, Zhejiang Province, China; d Department of Biomedical Sciences and One Health Center for Zoonoses and Tropical Veterinary Medicine, Ross University School of Veterinary Medicine, Basseterre, St. Kitts and Nevis; e Department of Veterinary Biosciences, Melbourne Veterinary School, The University of Melbourne, Parkville, Victoria, Australia; Weill Cornell Medicine

**Keywords:** *Toxoplasma gondii*, PP2Acα, B′/PR61 subunit, amylopectin metabolism, virulence

## Abstract

Here, we report that the inhibition of the PP2A subfamily by okadaic acid results in an accumulation of polysaccharides in the acute infection stage (tachyzoites) of Toxoplasma gondii, which is a protozoan of global zoonotic importance and a model for the apicomplexan parasites. The loss of the catalytic subunit α of PP2A (Δ*PP2Acα*) in RHΔ*ku80* leads to the polysaccharide accumulation phenotype in the base of tachyzoites as well as residual bodies and significantly compromises the intracellular growth *in vitro* and the virulence *in vivo*. A metabolomic analysis revealed that the accumulated polysaccharides in Δ*PP2Acα* are derived from interrupted glucose metabolism, which affects the production of ATP and energy homeostasis in the T. gondii knockout. The assembly of the PP2Acα holoenzyme complex involved in the amylopectin metabolism in tachyzoites is possibly not regulated by LCMT1 or PME1, and this finding contributes to the identification of the regulatory B subunit (B′/PR61). The loss of B′/PR61 results in the accumulation of polysaccharide granules in the tachyzoites as well as reduced plaque formation ability, exactly the same as Δ*PP2Acα*. Taken together, we have identified a PP2Acα-B′/PR61 holoenzyme complex that plays a crucial role in the carbohydrate metabolism and viability in T. gondii, and its deficiency in function remarkably suppresses the growth and virulence of this important zoonotic parasite both *in vitro* and *in vivo*. Hence, rendering the PP2Acα-B′/PR61 holoenzyme functionless should be a promising strategy for the intervention of *Toxoplasma* acute infection and toxoplasmosis.

**IMPORTANCE** Toxoplasma gondii switches back and forth between acute and chronic infections, mainly in response to host immunologic status, which is characterized by flexible but specific energy metabolism. Polysaccharide granules are accumulated in the acute infection stage of T. gondii that have been exposed to a chemical inhibitor of the PP2A subfamily. The genetic depletion of the catalytic subunit α of PP2A leads to this phenotype and significantly affects the cell metabolism, energy production, and viability. Further, a regulatory B subunit PR61 is necessary for the PP2A holoenzyme to function in glucose metabolism and in the intracellular growth of T. gondii tachyzoites. A deficiency of this PP2A holoenzyme complex (PP2Acα-B′/PR61) in T. gondii knockouts results in the abnormal accumulation of polysaccharides and the disruption of energy metabolism, suppressing their growth and virulence. These findings provide novel insights into cell metabolism and identify a potential target for an intervention against a T. gondii acute infection.

## INTRODUCTION

In eukaryotes, nearly half of all proteins undergo phosphorylation/dephosphorylation on serine, threonine, and/or tyrosine residues, representing one of the most important posttranslational modifications of whole biological processes (1). Interestingly, the protein phosphatome, the number and composition of phosphatases that exert dephosphorylation activity on proteins, varies dramatically among different organisms, particularly between eukaryotic pathogens and their hosts. For instance, in contrast to them being only about 30% of the protein phosphatases in humans, serine/threonine (Ser/Thr) phosphatases account for approximately 80% of the protein phosphatome of Toxoplasma gondii, which is the most successful intracellular protozoan parasite, affecting almost all warm blooded animals and one-third world population (2, 3). Ser/Thr phosphatase activities are mostly accomplished by protein phosphatase 1 (PP1) and protein phosphatase 2A (PP2A) in many eukaryotes, and these enzymes catalyze more than 90% of Ser/Thr phosphorylated protein reactions (4). However, although great efforts have been made toward elucidating the roles of Ser/Thr phosphatases in T. gondii and in other apicomplexan parasites (5, 6), there is still a lack of comprehensive functional information on PP1 and PP2A in these important pathogens.

The chemical inhibitors of phosphatases have been widely used to analyze their functions in human cells *in vitro* (7, 8) and occasionally in apicomplexan parasites (9). Recently, in our high-throughput screening of the essential phosphatases of T. gondii, using natural product inhibitors (unpublished data), we have observed a remarkable accumulation of amylopectin in the tachyzoites (the acute infection stage) that have been exposed to okadaic acid (an inhibitor for PP2A and PP1) (10) *in vitro*, which is usually accumulated as a long-term carbon source in the bradyzoites (the chronic infection stage) and oocysts (11–13). Molecules involved in carbon metabolism, such as Ca^2+^-dependent protein kinase (CDPK2), fructose 1,6-bisphosphatase 2 (FBP2), and glycogen phosphatase (GP), have been reported to play critical roles in T. gondii (14–16). The conditional knockdown of *Tg*FBP2 results in the complete loss of intracellular growth *in vitro* under glucose-replete conditions as well as the loss of acute virulence in mice (16), whereas a deficiency of CDPK2 or GP has been reported to inhibit the formation of cysts (14, 15). Therefore, the okadaic acid treatment-induced amylopectin accumulation in the tachyzoites of T. gondii strongly indicates a crucial role of PP2A in the carbohydrate metabolism and, possibly, the intracellular survival of this zoonotic pathogen in hosts.

In this study, using an integrative approach, we elucidated that the holoenzyme consisting of the catalytic subunit α, scaffolding subunit A, and regulatory subunit B′/PR61 of PP2A determined the biosynthesis and degradation of polysaccharide granules in T. gondii tachyzoites. A genetic deficiency of this holoenzyme resulted in the accumulation of amylopectin, which was likely derived from glucose, rather than glutamine, in the tachyzoites of T. gondii and markedly compromised the intracellular proliferation of T. gondii tachyzoites *in vitro* as well as their virulence *in vivo* in mice. These results provide novel insights into the role of Ser/Thr phosphatases in carbon metabolism as well as the associated regulatory mechanisms during the acute and latent infections of toxoplasmosis in hosts, which paves an exciting new way for new drug/vaccine discovery.

## RESULTS

### PP2Acα is necessary for the normal polysaccharide metabolism in T. gondii tachyzoites.

**FIG 1:**
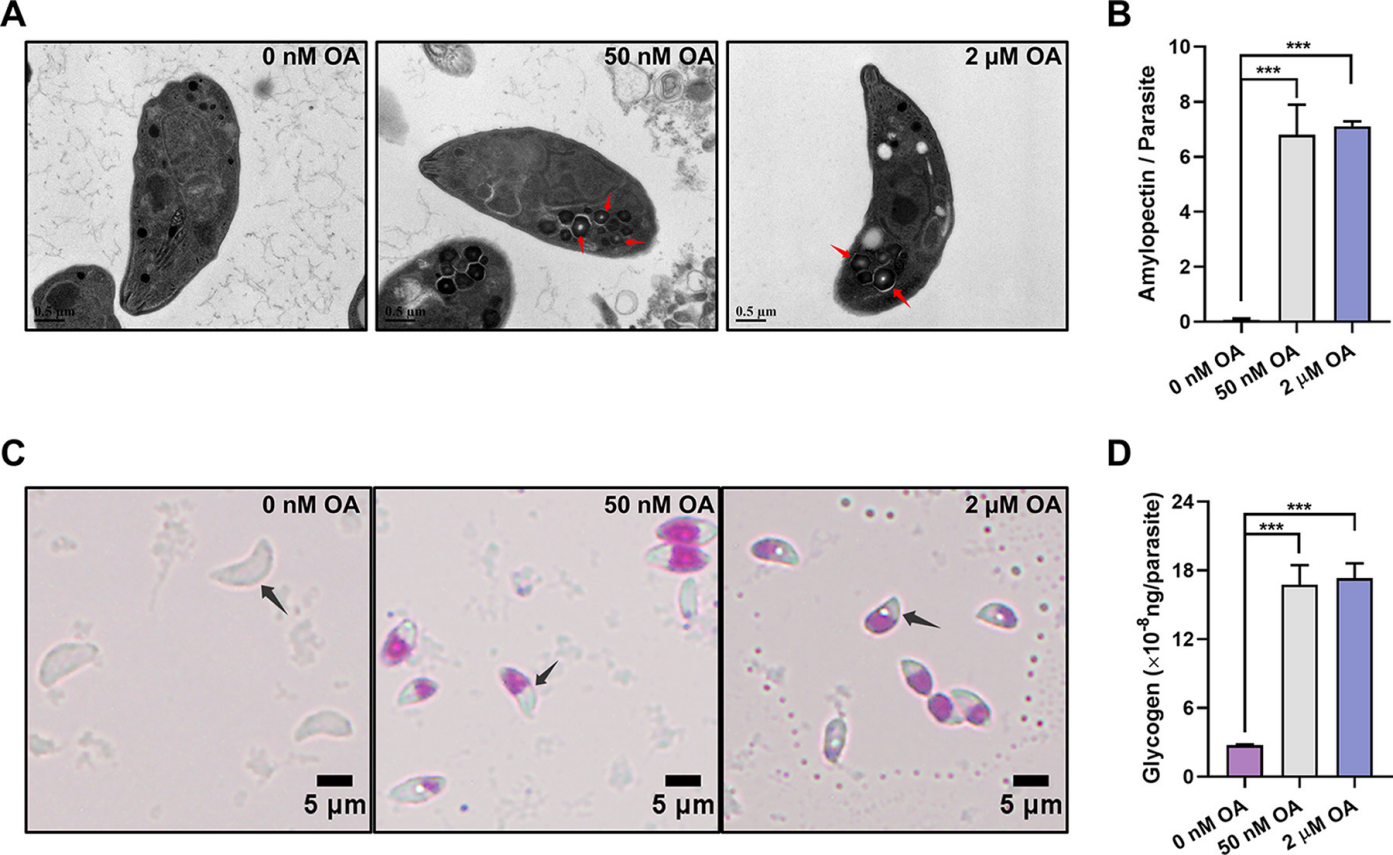
Okadaic acid (OA) treatment leads to the presence of polysaccharide granules in *Toxoplasma* tachyzoites. (A) Transmission electron microscopy of T. gondii tachyzoites treated with 0 nM, 50 nM, or 2 μM OA for 3 h. Semicrystalline granular deposits are indicated by red arrows. (B) The numbers of polysaccharide granules observed in the tachyzoites treated with 0 nM, 50 nM, or 2 μM OA. At least 50 tachyzoites are analyzed in each group. The data are presented as the mean ± standard deviation (SD). (C) Periodic acid-Schiff staining of tachyzoites treated with 0 nM, 50 nM, or 2 μM OA. Tachyzoites are indicated by black arrows. (D) Quantification of glycogen in tachyzoites treated with 0 nM, 50 nM, or 2 μM OA. The data are presented as the mean ± standard deviation (SD). ***, *P ≤ *0.001, by a *t* test.

Using transmission electron microscopy, we observed semicrystalline granules at the basal end of T. gondii tachyzoites that had been exposed to 50 nM or 2 μM okadaic acid (OA) for 3 h *in vitro* (Fig. 1A and B). Periodic acid-Schiff staining, which is a method used to detect polysaccharides (16), of the treated extracellular tachyzoites (exposed to 50 nM or 2 μM OA for 3 h) showed an accumulation of polysaccharides (Fig. 1C and D). Neither amylopectin granules nor polysaccharides were detected in the control tachyzoites, and the accumulation of amylopectin in the OA-treated groups did not multiply with the increase of the OA treatment concentration. Considering the specific potencies of OA to inhibit both PP1 and PP2A in mammalian cells (17) and the previous report that 50 nM OA did not obviously inhibit recombinant *Tg*PP1 (18), our results indicate that the PP2A subfamily plays a role in the metabolism of polysaccharides of T. gondii tachyzoites.

**FIG 2:**
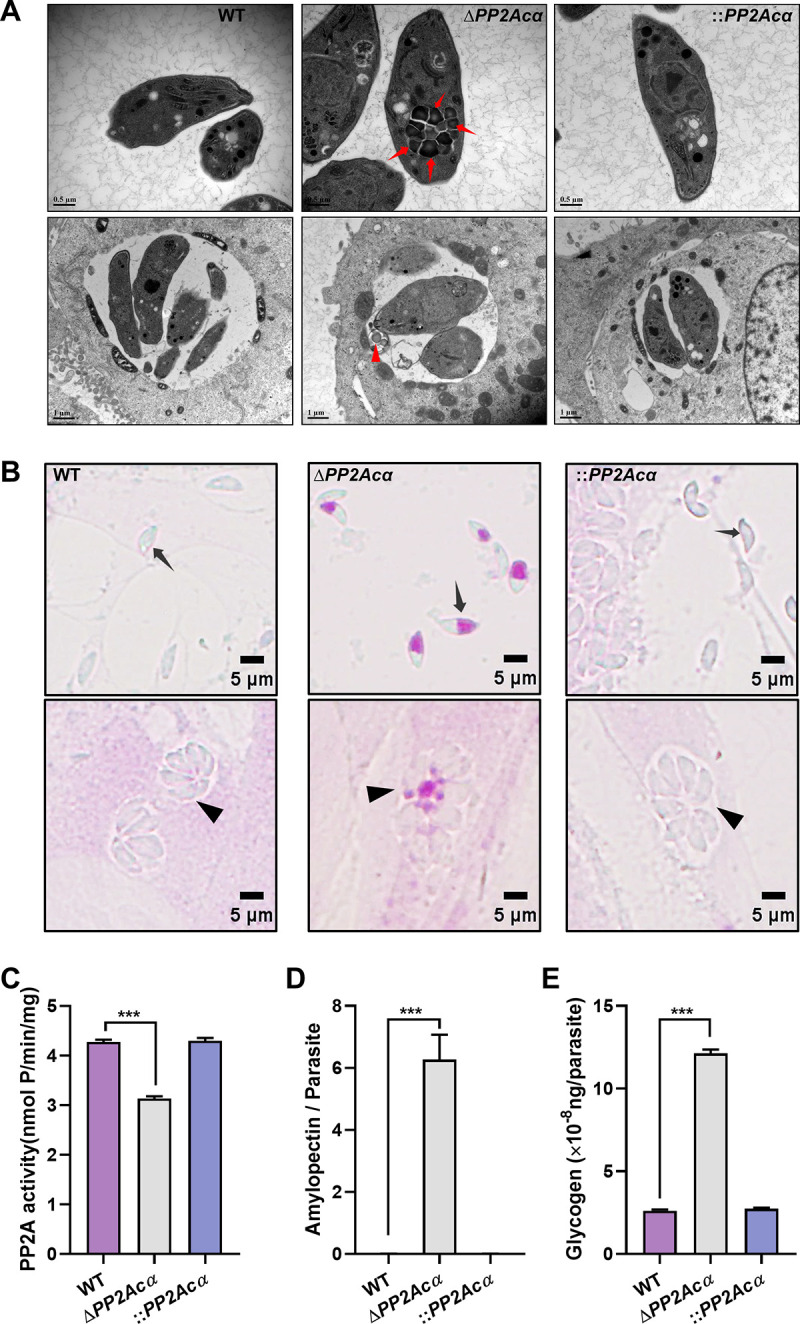
PP2Acα deficiency leads to the presence of polysaccharide granules in *Toxoplasma* tachyzoites. (A) Transmission electron microscopy of WT, Δ*PP2Acα*, and ::*PP2Acα* tachyzoites isolated from HFF-1 cells (top panels) and replicated within HFF-1 cells (bottom panels). Semicrystalline granular deposits are indicated by red arrows (top panel) or red arrowheads (bottom panel). (B) Periodic acid-Schiff staining of WT, Δ*PP2Acα*, and ::*PP2Acα* tachyzoites escaped from HFF-1 cells (top panels) and replicated within HFF-1 cells (bottom panels). Tachyzoites or parasitophorous vacuoles are indicated by black arrows and arrowheads, respectively. (C) The activity of PP2A in wild-type (WT), *PP2Acα* knockout (Δ*PP2Acα*), and its complemented (::*PP2Acα*) cells of T. gondii. The data are presented as the mean ± standard deviation (SD) from three independent technical repeats. (D) The numbers of polysaccharide granules observed in WT, Δ*PP2Acα*, and ::*PP2Acα* tachyzoites escaped from HFF-1 cells. At least 50 tachyzoites are analyzed in each group. The data are presented as the mean ± standard deviation (SD). (E) Quantification of glycogen in WT, Δ*PP2Acα*, and ::*PP2Acα* cells. The data are presented as the mean ± standard deviation (SD). ***, *P ≤ *0.001, by a *t* test.

To confirm the role of the PP2A subfamily in polysaccharide metabolism, we performed the individual genetic deletion of the catalytic subunit of PP2Acα (TGGT1_224220), PP2Acβ (TGGT1_215170), PP4c (TGGT1_286210), or PP6c (TGGT1_301010) (19) in T. gondii. Although the latter three were not efficiently knocked out, *PP2Acα* knockout cells (Δ*PP2Ac*α) were successfully obtained (Fig. S1A and B). The PP2A activity in the knockout tachyzoites was determined by measuring the dephosphorylation of PKpTIRR, which showed three quarters of the overall activity of PP2A in the wild-type (WT) tachyzoites (*P < *0.001) (Fig. 2C). Both semicrystalline granule and polysaccharide accumulation were detected in the *PP2Acα* knockout cells, similar to those observed in the OA-treated extracellular tachyzoites (Fig. 2A and B). In addition, polysaccharide staining was also observed in the residual bodies of the intracellular Δ*PP2Ac*α tachyzoites (Fig. 2A and B). Compared with WT parental controls, the numbers of amylopectin granules (*P < *0.001) and the levels of glycogen (*P < *0.001) in the knockout tachyzoites were increased more than 100 times and approximately 5 times, respectively. (Fig. 2D and E). These defective phenotypes were completely rescued by reintroducing the *PP2Acα* gene back into Δ*PP2Ac*α T. gondii (::*PP2Acα*) (Fig. 2A and E; Fig. S1C and D). Collectively, these findings unequivocally showed that PP2Acα plays a crucial role in the normal metabolism of amylopectin in T. gondii and that a deficiency of this process in tachyzoites is likely to affect their survival or virulence in hosts.

### PP2Acα deficiency compromises the intracellular growth and virulence of T. gondii tachyzoites.

**FIG 3:**
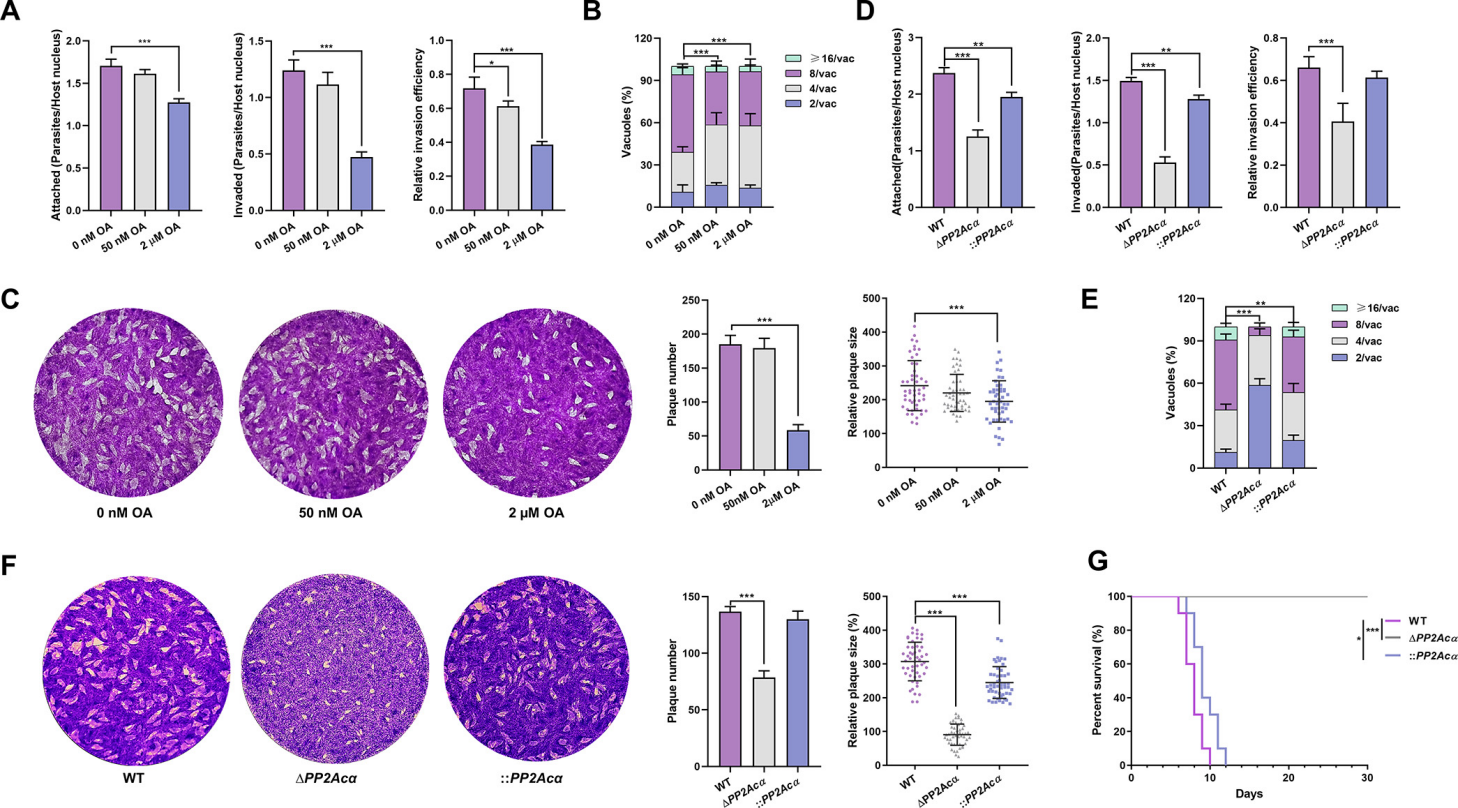
PP2Acα deficiency compromises the intracellular growth and virulence of Toxoplasma gondii tachyzoites. (A) Newly isolated tachyzoites were treated with 0 nM, 50 nM, or 2 μM okadaic acid (OA). Their attachment, invasion, and invasion efficiency into HFF-1 cells were assayed. *, *P ≤ *0.05; ***, *P ≤ *0.001, all by *t* tests. (B) The numbers of tachyzoites per parasitophorous vacuole in HFF-1 cells as an indicator of intracellular replication followed by OA treatment, as described in panel A. At least 50 vacuoles are analyzed for each group. ***, *P ≤ *0.001, by a two-way ANOVA. (C) The numbers and areas of plaques formed by 0 nM, 50 nM, or 2 μM OA-treated tachyzoites. ***, *P ≤ *0.001, both by *t* tests. (D) The attachment, invasion, and invasion efficiency of the tachyzoites of wild-type (WT), *PP2Acα* knockout (Δ*PP2Acα*), and its complemented (::*PP2Acα*) cells to HFF-1 cells. **, *P ≤ *0.01; ***, *P ≤ *0.001, all by *t* tests. (E) The numbers of tachyzoites per parasitophorous vacuole in HFF-1 cells of WT, Δ*PP2Acα*, and ::*PP2Acα* tachyzoites. At least 50 vacuoles were analyzed for each mutant. **, *P ≤ *0.01; ***, *P ≤ *0.001, by a two-way ANOVA. (F) The numbers and areas of plaques formed by WT, Δ*PP2Acα*, and ::*PP2Acα* tachyzoites. ***, *P ≤ *0.001, both by *t* tests. (G) The survival rate of ICR mice infected with WT, Δ*PP2Acα*, and ::*PP2Acα* tachyzoites (100 tachyzoites per mouse, 10 mice per group). *, *P ≤ *0.05; ***, *P ≤ *0.001.

In order to verify the crucial roles of *PP2Acα* within T. gondii, we assessed the intracellular growth and virulence of Δ*PP2Ac*α T. gondii tachyzoites. After treatment with 50 nM OA for 1 h *ex vivo*, the RHΔ*ku80* tachyzoites were used to infect HFF-1 cells for 20 min, and the number of tachyzoites that adhered to (labeled with Alexa Fluor 488) and invaded (only labeled with Alexa Fluor 488 but not Alexa Fluor 594) cells showed no significant difference. After treatment with 50 nM OA for 3 h *ex vivo*, the RHΔ*ku80* tachyzoites were used to infect HFF-1 cells that were cultured with normal cell medium for 7 days, and the numbers and areas of the plaques formed by the tachyzoites showed no significant difference. However, their intracellular replication was significantly affected when cultured in medium supplemented with 50 nM OA. The parasitophorous vacuoles containing more than 4 tachyzoites each were reduced from 60% of the controls to 40% (*P < *0.001) after invading cells for 24 h (Fig. 3A–C). This may be associated with carbohydrate metabolism (including, but likely not limited to polysaccharide accumulation), and its interruption has been reported to be harmful to this parasite (16, 20–23). However, tachyzoites exposed to 2 μM OA significantly decreased their ability to adhere to and invade host cells, which is consistent with the results of previous reports (24). The effect of PP2Acα deficiency on the lytic cycle (adhesion, invasion, replication, and escape) of T. gondii was determined by adhesion and invasion, intracellular replication, and plaque assays in the knockout tachyzoites. Interestingly, the adhesion and invasion efficiency of *PP2Acα* knockout tachyzoites *in vitro* were reduced by approximately 50% and 40%, respectively (*P < *0.01) (Fig. 3D), as well as intracellular replication and the ability to form plaques (Fig. 3E and F). Remarkably, ICR mice infected with Δ*PP2Ac*α tachyzoites (100 per mouse) all survived 30 days postinfection, whereas all those inoculated with wild-type tachyzoites died within 10 days postinfection (Fig. 3G; Fig. S1E). Further, the mice infected with the add-back cells (i.e., by reintroducing the *PP2Acα* gene back into the knockout T. gondii), all died within 12 days postinfection, as well (Fig. 3G). These results clearly showed that *PP2Acα* plays an essential role in the virulence of T. gondii in mice, which may be related to the inhibition of extensive dephosphorylation (4), resulting in decreases in the abilities of invasion and proliferation.

### PP2Acα plays a crucial role in the energy metabolism and cell metabolism in T. gondii.

**FIG 4:**
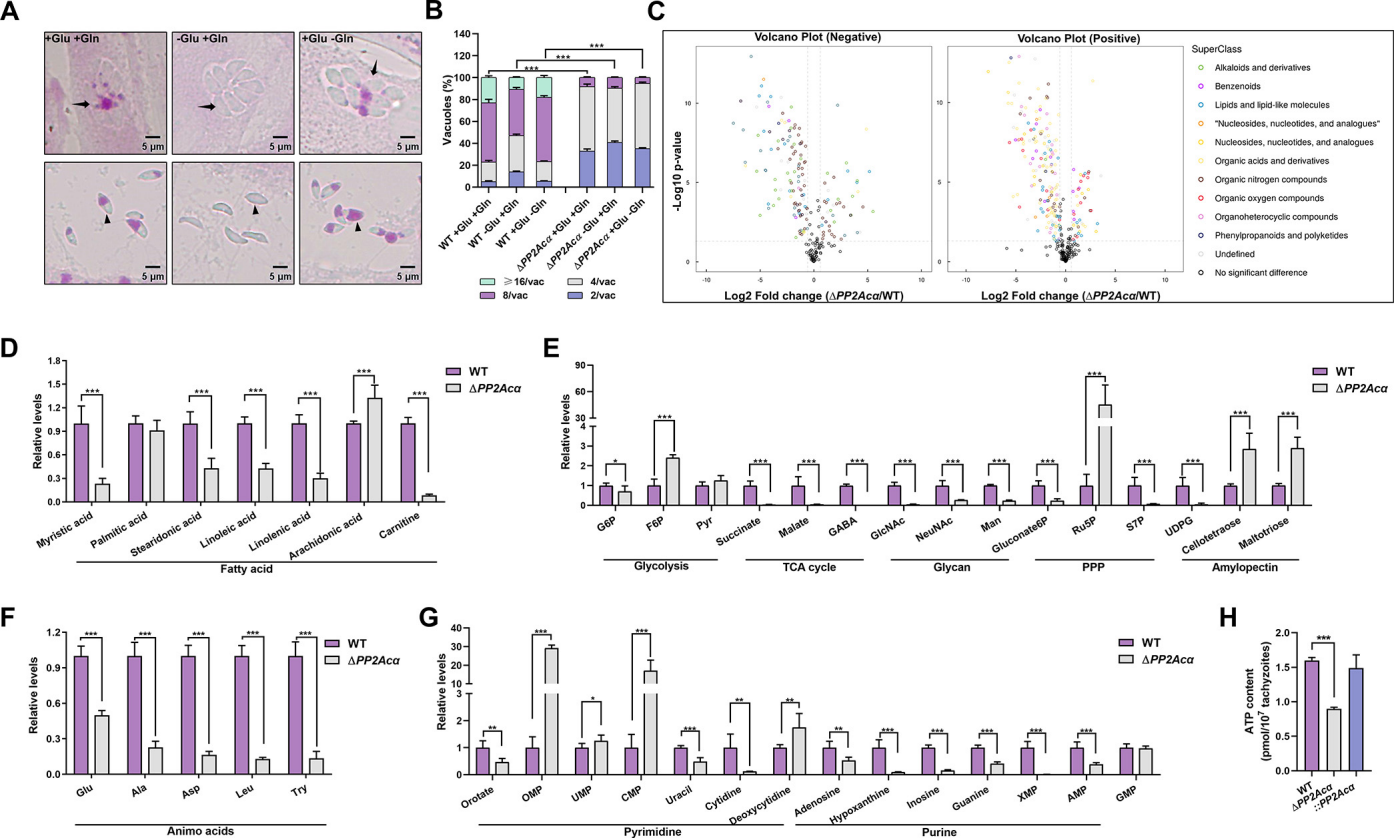
PP2Acα plays a crucial role in the energy metabolism and cell metabolism in Toxoplasma gondii. (A) Periodic acid-Schiff staining. *PP2Acα* knockout (Δ*PP2Acα*) tachyzoites replicated within (top panel) or escaped from HFF-1 cells (bottom panel) cultured in DMEM (control) or medium in the absence of glucose (−Glu) or glutamine (−Gln). Parasitophorous vacuoles and tachyzoites are indicated by black arrows and arrowheads, respectively. (B) Intracellular replication per number of tachyzoites per vacuole of WT or Δ*PP2Acα* cultured in medium (+Glu and +Gln), or medium in the absence of glucose (−Glu) or glutamine (−Gln). ***, *P ≤ *0.001, by a two-way ANOVA. (C) Metabolomic differences between Δ*PP2Acα* and WT tachyzoites, identified by negative and positive ionization polarity. The superclass of metabolites significantly changed between Δ*PP2Acα* and WT tachyzoites, and these changes are indicated in the volcano plots and color coded. (D–G) Relative levels of amino acids, purine-, pyrimidine-, fatty acids-, glycolysis-, tricarboxylic acid (TCA)-, glycan-, pentose phosphate (PPP)- and amylopectin-associated metabolites between Δ*PP2Acα* and WT tachyzoites. *, *P ≤ *0.05; **, *P ≤ *0.01; ***; *P ≤ *0.001, all by multiple *t* tests. (H) ATP content in WT, Δ*PP2Acα*, and its complemented (::*PP2Acα*) tachyzoites. The data are the mean ± standard deviation (SD) of three independent repeats. ***, *P ≤ *0.001, by a *t* test.

Having demonstrated the involvement of *PP2Acα* in amylopectin metabolism and in the proliferation of tachyzoites of T. gondii, we attempted to identify the carbon source of the accumulated amylopectin by assessing the accumulation of polysaccharides in the intracellular and extracellular *PP2Acα* knockout tachyzoites after depleting glucose or glutamine in their culture media, as they are important carbon sources for the parasite (25, 26). In the absence of glucose, no polysaccharide staining was found in the intracellular or extracellular tachyzoites of Δ*PP2Ac*α T. gondii. In contrast, the amylopectin accumulation remained in the knockout tachyzoites without glutamine (Fig. 4A). It is known that the accumulation of certain intermediate products in carbohydrate metabolism affects the intracellular growth and reproduction of tachyzoites (27). Therefore, we further assessed the intracellular replication of Δ*PP2Ac*α after removing one of the two carbon sources from the culture medium, and we found that the growth inhibition that was caused by PP2Acα deletion could not be rescued (Fig. 4B). These results elucidated that the carbon source that was responsible for the accumulation of polysaccharide granules in Δ*PP2Ac*α tachyzoites is glucose, rather than glutamine, and the growth inhibition that is caused by PP2Acα deficiency is not induced by the accumulation of the toxic intermediate products in carbohydrate metabolism.

In order to provide an overview of the PP2Acα-associated cell metabolism and energy homeostasis in T. gondii, the metabolites in the wild-type and knockout tachyzoites were analyzed. Metabolomic profiling showed that PP2Acα deficiency affected multiple processes, including the tricarboxylic acid (TCA) cycle, pentose phosphate pathway (PPP), and fatty acid metabolism, compared with those in wild-type tachyzoites (Fig. 4C–G). The abundance of intermediate products of carbohydrate metabolism, such as malate, succinate, gamma aminobutyric acid (GABA), sedoheptulose-7-phosphate (S7P), and UDP glucose (UDPG), which are the intermediate products of glucose metabolism, decreased significantly, and the content of fatty acid synthesis products decreased, as well as the abundance of important amino acids. These results clearly indicated a metabolic disturbance in the energy metabolism and nucleotide metabolism of Δ*PP2Ac*α tachyzoites. Therefore, we further measured the ATP content in the wild-type and Δ*PP2Acα* tachyzoites and found the ATP level decreased by approximately 50% in the knockout T. gondii (*P < *0.001) (Fig. 4H). The reduced ATP production in Δ*PP2Ac*α was rescued by introducing the *PP2Acα* back (Fig. 4H). As glycolysis and oxidative phosphorylation are the main sources of intracellular ATP, we speculated that a deficiency of PP2Acα may inhibit key factors of these pathways leading to a decrease in ATP levels and widespread metabolic disturbances as well as an increase in glucose influx into the glycogen synthesis pathway, thereby resulting in the accumulation of amylopectin. These results confirmed that PP2Acα plays a crucial role in the energy metabolism and cell metabolism in T. gondii.

### The role of PP2Acα in the glucose metabolism of tachyzoites is not regulated by leucine carboxyl methyl transferase 1 (LCMT1) or by protein phosphatase methylesterase 1 (PME1).

**FIG 5:**
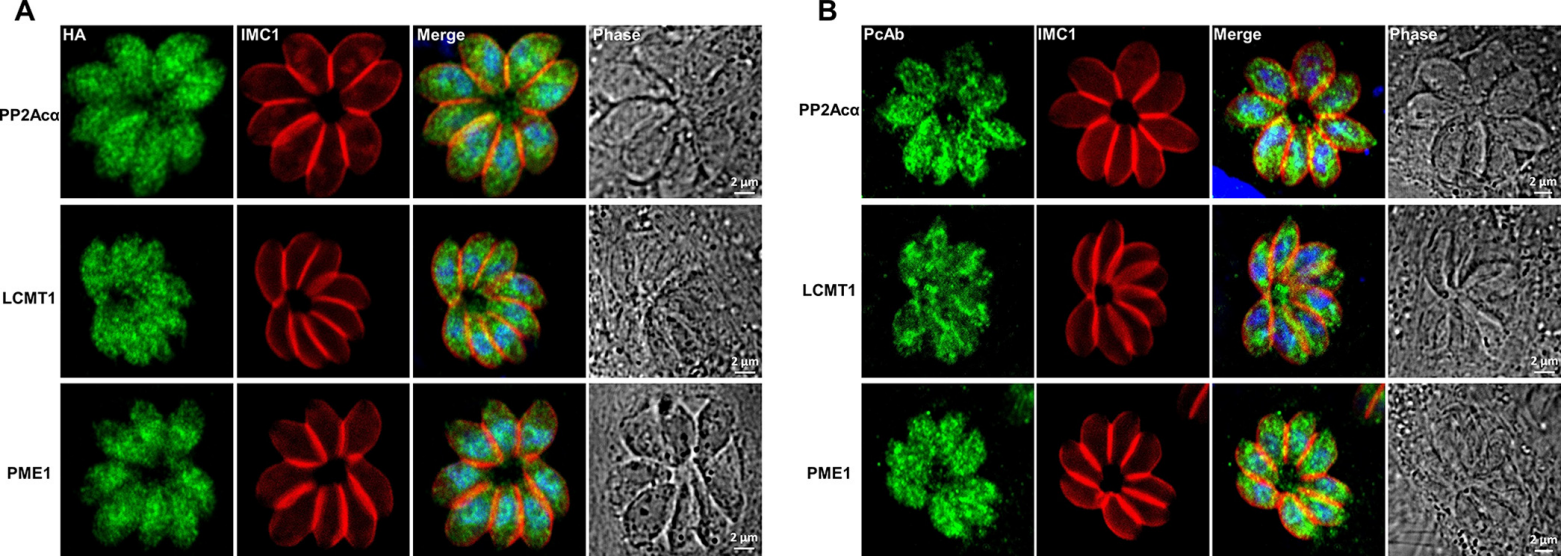
Subcellular distributions of PP2Acα and its methylation regulators in Toxoplasma gondii tachyzoites. (A) Endogenous 3× hemagglutinin (HA)-tagged PP2Acα, leucine carboxyl methyl transferase 1 (LCMT1), and protein phosphatase methylesterase 1 (PME1) in the cytoplasm and nuclei of tachyzoites are indicated in green. The distribution of the inner membrane complex (IMC1) is indicated in red. The nuclei were stained by 4′,6-diamidino-2′-phenylindole (DAPI) (blue). (B) The distributions of PP2Acα, LCMT1, and PME1 in the cytoplasm and nuclei of tachyzoites are indicated by a polyclonal antibodies-based immunofluorescence assay (green). The distribution of the IMC1 and nuclei are indicated in red and blue, respectively.

**FIG 6:**
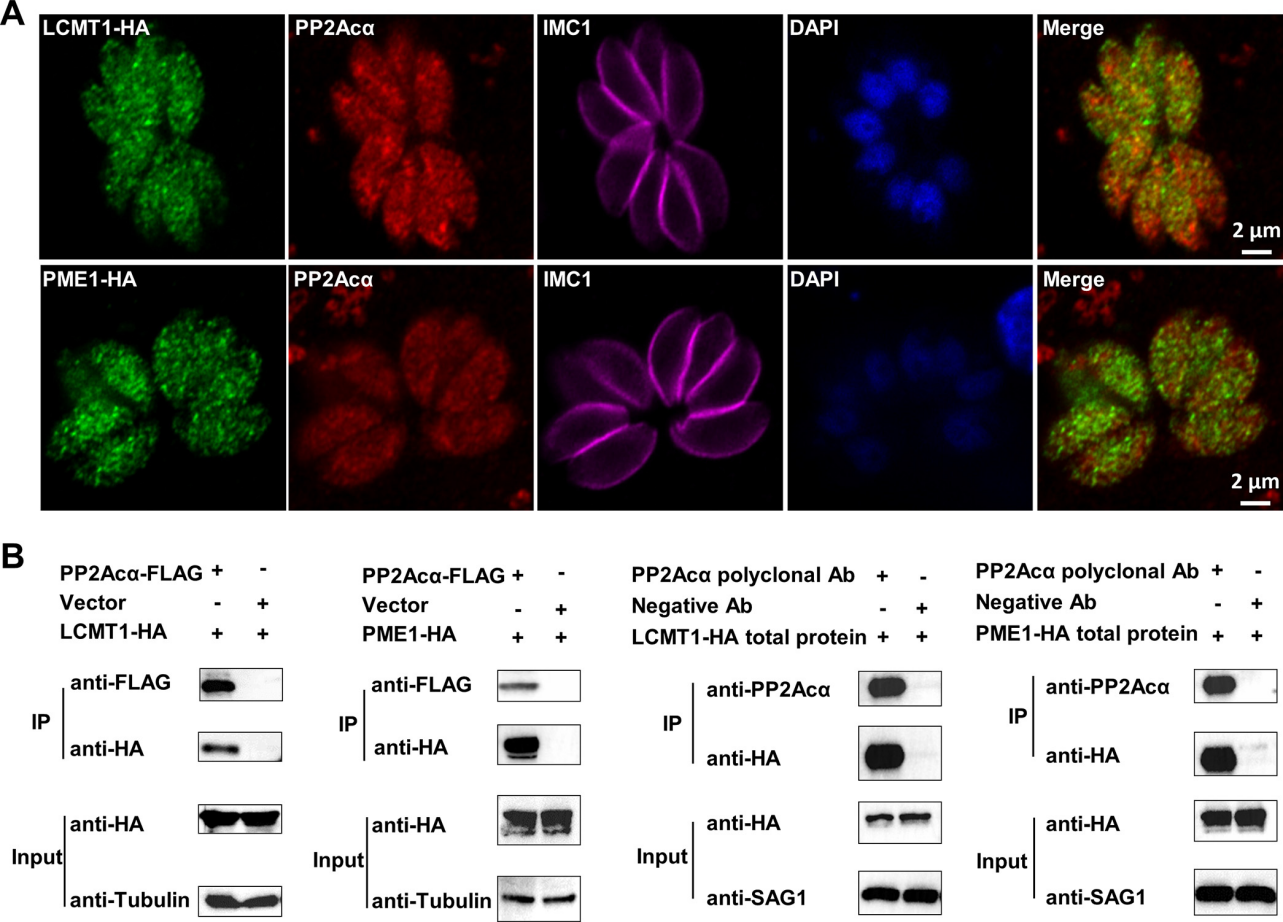
PP2Acα colocalizes with methylation regulators. (A) Subcellular distributions of PP2Acα (red), leucine carboxyl methyl transferase 1 (LCMT1; green), and protein phosphatase methylesterase 1 (PME1; green) in Toxoplasma gondii tachyzoites. The distribution of the inner membrane complex (IMC1) and nuclei are indicated in red and blue, respectively. (B) Coimmunoprecipitation of FLAG-tagged PP2Acα and HA-tagged methylation regulators isolated from either HEK 293T cells (the left two panels) or tachyzoites (the right two panels). Cellular lysates of HEK 293T cells or tachyzoites (input) are incubated with anti-Flag affinity gel or protein A/G magnetic beads that had previously been incubated with PP2Acα polyclonal antibodies. Anti-FLAG/HA/tubulin/SAG1 antibodies are used in the Western blot analyses.

Considering that the PP2A holoenzyme is a heterotrimeric complex that contains the structural subunit A, regulatory subunit B, and catalytic subunit C (28), we were keen to identify the specific complex, especially the B subunit, that is involved in the glucose metabolism and energy metabolism in the acute infection stage of T. gondii. It has been elucidated that the binding of the PP2A core enzyme (A/C heterodimer) to certain B subunits can be regulated by the reversible methylation of the catalytic subunit via LCMT1 (TGGT1_237570) and PME1 (TGGT1_262140) (29, 30). T. gondii has both methylation regulatory factors, suggesting that such regulatory mechanisms may be retained in *Toxoplasma* (19). Therefore, we first aimed to determine the roles of LCMT1 and PME1 in the activity of PP2Acα and in the glucose metabolism of T. gondii tachyzoites. All of these proteins were detected in the cytoplasm and nuclei of tachyzoites in an immunofluorescence assay (IFA) (Fig. 5A) using antibodies to hemagglutinin tag endogenous 3× hemagglutinin-tagged PP2Acα, LCMT1, and PME1 (PP2Acα-3HA, 42 kDa; LCMT1-3HA, 54 kDa; and PME1-3HA, 53 kDa, respectively) in T. gondii (Fig. S2A and B). These subcellular localizations of PP2Acα, LCMT1, and PME1 in tachyzoites were confirmed in IFA using polyclonal antibodies against these proteins, respectively (Fig. S2C; Fig. 5B). Although the localization shown by some polyclonal antibodies is not exactly consistent with the localization indicated by those that are endogenously labeled, especially for LCMT1, this may be related to the insufficient specificity of the antibodies that we prepared (Fig. S2C). However, the localization patterns in both the cytoplasm and the nuclei were consistent with those of endogenously labeled proteins, supporting the accuracy of the endogenously labeled localization. Fig. 5A showed that PP2Acα and LCMT1 were distributed diffusely in the cytoplasm and the nuclei, whereas PME1 was also distributed in the cytoplasm but mostly accumulated in the nuclei. Then, we investigated the interactions between PP2Acα and the methylation regulators via both IFA and coimmunoprecipitation (Co-IP). IFA clearly revealed the partial colocalizations of PP2Acα and LCMT1 as well as PP2Acα and PME1 (Fig. 6A). Their relationships were further verified via Co-IP, using both HEK 293T cells and T. gondii tachyzoites (Fig. 6B). These results unequivocally indicated interactions between PP2Acα and the methylation regulators LCMT1 and PME1, which likely determine the architecture and activity of the PP2A holoenzyme complex in the tachyzoites of T. gondii.

**FIG 7:**
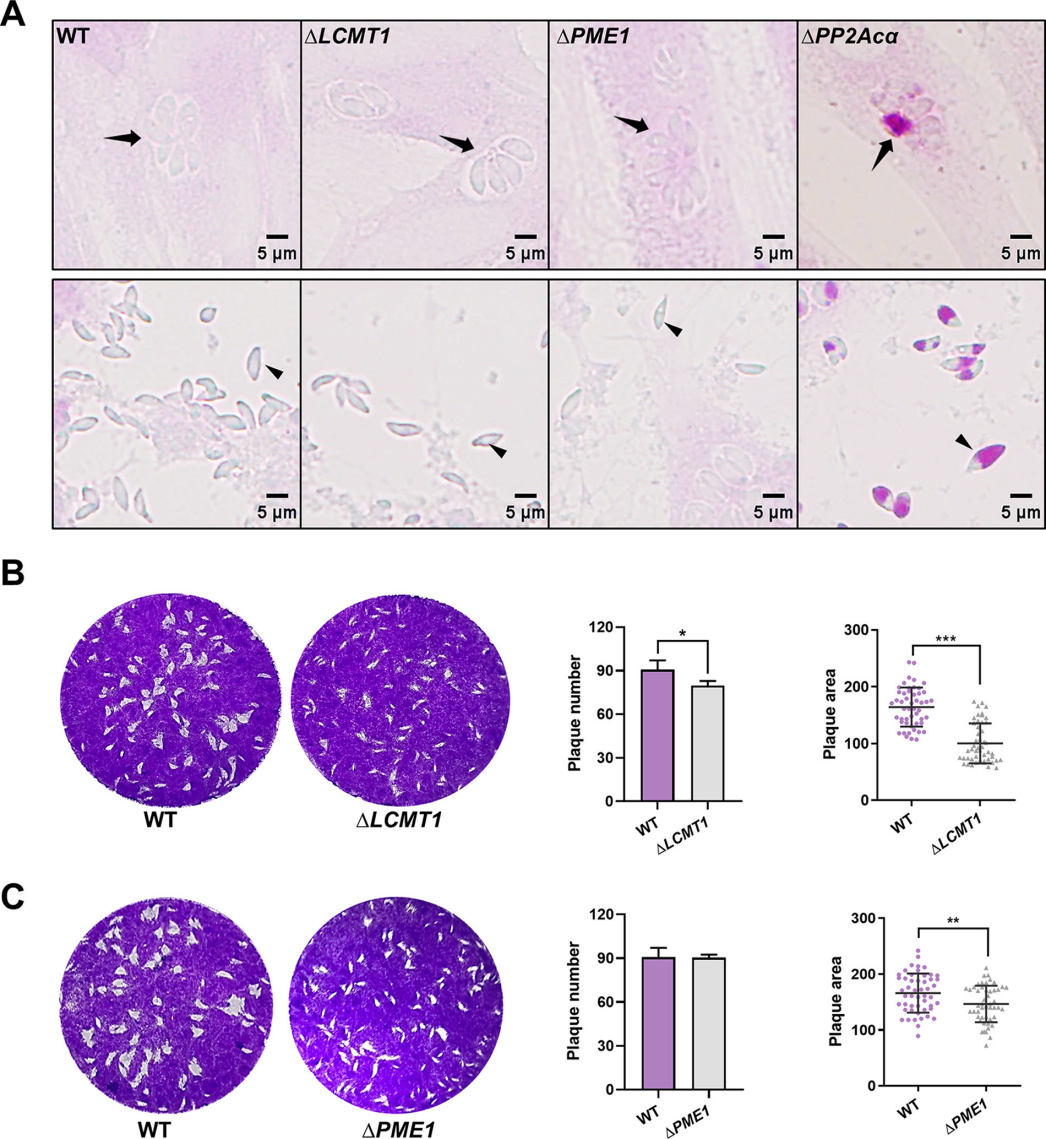
PP2Acα does not need a methylation-dependent regulatory subunit to control glucose metabolism in Toxoplasma gondii tachyzoites. (A) Periodic acid-Schiff (PAS) staining. Wild-type (WT), leucine carboxyl methyl transferase 1 (*LCMT1*) knockout (Δ*LCMT1*), protein phosphatase methylesterase 1 (*PME1*) knockout (Δ*PME1*), and *PP2Acα* knockout (Δ*PP2Acα*) tachyzoites replicated within HFF-1 cells (top panels) and escaped from HFF-1 cells (bottom panels). Parasitophorous vacuoles and tachyzoites are indicated by black arrows and arrowheads, respectively. (B and C) The numbers and areas of the plaques formed by WT, Δ*LCMT1*, or Δ*PME1* tachyzoites. *, *P ≤ *0.05; **, *P ≤ *0.01; ***, *P ≤ *0.001, all by *t* tests.

To confirm the regulatory roles of LCMT1 and PME1 in the PP2A holoenzyme regulating amylopectin metabolism, we generated LCMT1 and PME1 (Δ*LCMT1*, Δ*PME1*) knockout mutants of T. gondii (Fig. S3A and B). If the PP2A holoenzyme complex that regulates amylopectin metabolism is methylation-dependent, then LCMT1 deletion will lead to a similar accumulation of amylopectin granules. Since PME1 plays roles in both inhibiting PP2A activity and protecting catalytic subunits from ubiquitin/proteasome degradation (31), the effect of its absence on amylopectin metabolism is difficult to predict. Surprisingly, polysaccharide granules were detected in the tachyzoites of neither Δ*LCMT1* nor Δ*PME1* (Fig. 7A), although a deficiency of LCMT1 or PME1 resulted in reduced numbers (*P > *0.05) and areas (*P < *0.001) of plaques formed by the knockout tachyzoites (Fig. 7B and C). These results suggest that LCMT1 and PME1 play a role in the growth of tachyzoites but do not affect amylopectin metabolism in this parasite.

### B′/PR61 is the regulatory subunit of the PP2Acα holoenzyme that is involved in amylopectin metabolism.

**FIG 8:**
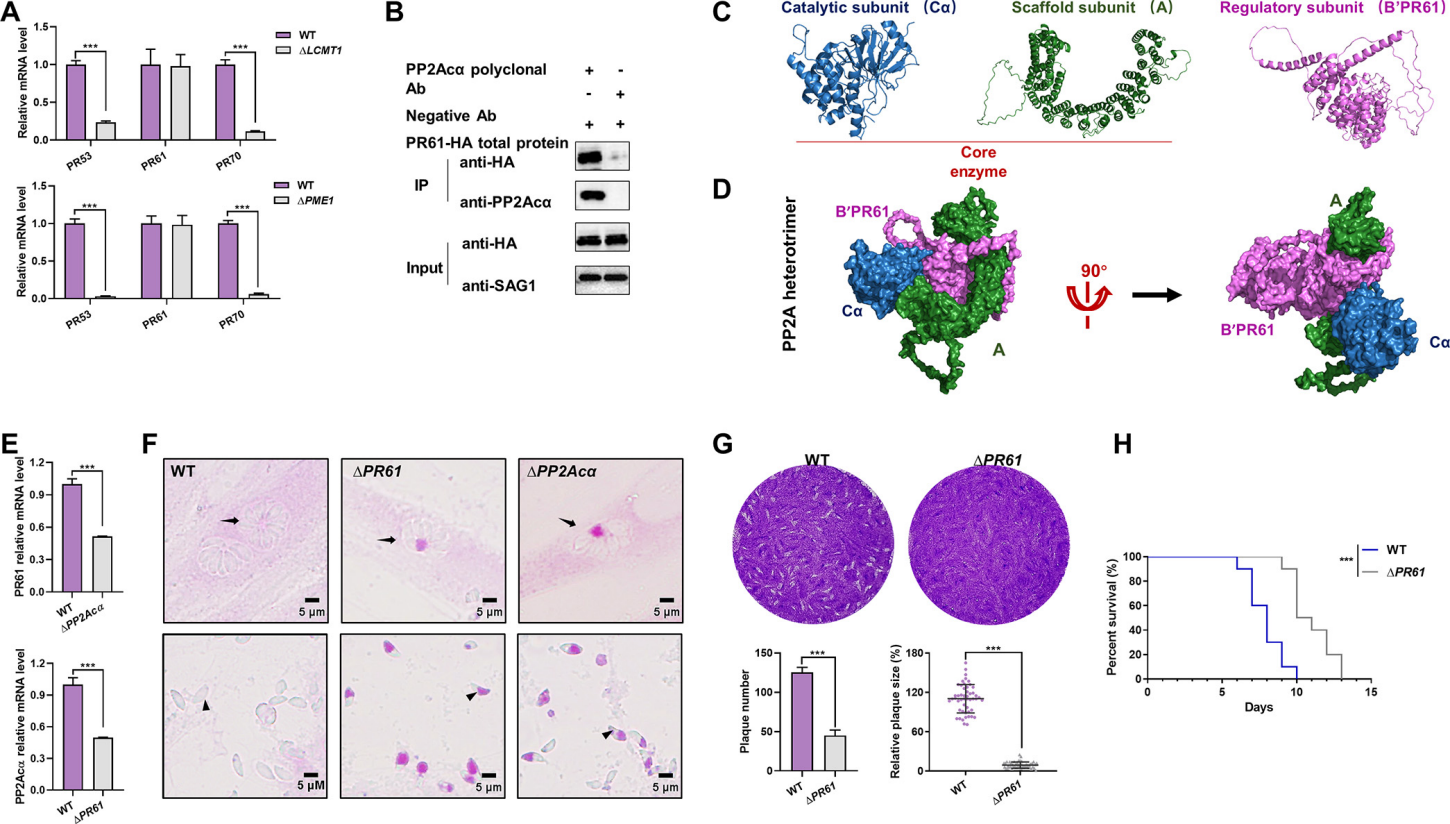
B′/PR61 is the regulatory subunit of the PP2Acα holoenzyme that regulates glucose metabolism in tachyzoites. (A) Transcriptional levels of B′/PR53, B′/PR61, and B′′/PR70 in the wild-type (WT), leucine carboxyl methyl transferase 1 (*LCMT1*) knockout (Δ*LCMT1*), and protein phosphatase methylesterase 1 (*PME1*) knockout (Δ*PME1*) tachyzoites of Toxoplasma gondii. The data are presented as the mean ± standard deviation (SD) of three independent repeats. ***, *P* ≤ 0.001, both by *t* tests. (B) Coimmunoprecipitation of PP2Acα and HA-tagged B′/PR61. The cellular lysates of the tachyzoites (input) were incubated with protein A/G magnetic beads that had previously been incubated with PP2Acα polyclonal antibodies. Anti-HA/SAG1 antibodies are used in the Western blot analyses. (C) Three-dimensional models of PP2Acα, B′/PR61, and A subunits of T. gondii, as predicted by AlphaFold2. PP2Acα combines with one end of subunit A to form the core enzyme. (D) *In silico*-modeled PP2Acα-A-B′/PR61 heterotrimer of T. gondii, with the subunits indicated by blue, green, and pink, respectively. (E) Transcriptional levels of the B′/PR61 subunit in wild-type (WT) as well as *PP2Acα* knockout (Δ*PP2Acα*) tachyzoites, and *vice versa*. The data are presented as the mean ± standard deviation (SD) of three independent repeats. ***, *P* ≤ 0.001, both by *t* tests. (F) Periodic acid-Schiff (PAS) staining. Wild-type (WT), *B′/PR61* knockout (Δ*PR61*), and Δ*PP2Acα* tachyzoites within HFF-1 cells (top panels) and escaped from HFF-1 cells (bottom panels). Parasitophorous vacuoles and tachyzoites are indicated by black arrows and arrowheads, respectively. (G) The numbers and areas of the plaques formed by the WT and Δ*PR61* tachyzoites. ***, *P ≤ *0.001, by a *t* test. (H) Survival rate of ICR mice infected with WT and Δ*PR61* tachyzoites, with 100 tachyzoites per mouse and 10 mice per group. ***, *P ≤ *0.001.

In total, three B subunits have been identified in T. gondii so far, which are PR53 (TGGT1_283720) of the B′ family (B′/PR53), B′/PR61 (TGGT1_246510), and B′′/PR70 (TGGT1_200400) (19). The transcriptional levels of B′/PR53 and B′′/PR70 were significantly decreased by more than 70% in both Δ*LCMT1* (*P < *0.001) and Δ*PME1* (*P < *0.001) knockout cells, whereas those of the B′/PR61 reminded unchanged, compared to those of parental wild-type controls (Fig. 8A). Therefore, B′/PR61 is likely the regulatory subunit of the PP2Acα holoenzyme involved in amylopectin metabolism.

The biochemical and structural relationship between PP2Acα and B′/PR61 was further investigated via Co-IP (Fig. 8B) and *in silico* protein-protein docking analyses (Fig. 8C and D). In addition, a B′/PR61 mutant (Δ*PR61*) of T. gondii was generated (Fig. S4A and B), in which the transcriptional level of PP2Acα decreased significantly and *vice versa* (*P < *0.001) (Fig. 8E), compared with controls. More importantly, just like Δ*PP2Acα*, Δ*PR61* resulted in the polysaccharide accumulation in the base of the tachyzoites and residual bodies (Fig. 8F), a reduction of plaque formation (Fig. 7G), and a reduction of tachyzoite virulence in mice (Fig. 8H), although there was less in magnitude in the virulence losses. These results confirmed that B′/PR61 is the regulatory subunit of the PP2Acα holoenzyme in the glucose metabolism in T. gondii.

## DISCUSSION

The apicomplexan protozoan T. gondii is an obligate intracellular parasite. Its infection in humans is of great concern in public health worldwide, particularly in pregnant women and children (32, 33). This parasite can reversibly switch between acute infection (tachyzoite) and chronic infection (bradyzoite) stages (13) in response to the host immune status to achieve immune evasion and sufficient parasitism (34, 35). Flexible and specialized metabolic networks have been reported between the tachyzoites and bradyzoites of T. gondii and other apicomplexan protozoa. For instance, T. gondii, coccidia, and *Cryptosporidium* spp. produce polysaccharide granules (in the form of plant-type amylopectin) as long-term energy sources reserved in unfavorable conditions (12, 36). These are barely present in fast-replicating tachyzoites, but they accumulate in bradyzoites during chronic infection and oocysts (11, 12). We have recently discovered a great amount of amylopectin accumulated in the tachyzoites of T. gondii that had been exposed to an inhibitor of PP1 and PP2A. Considering that carbon metabolism in T. gondii has been proposed as a promising target for a novel intervention (37, 38), in the current study, we elucidated the molecular mechanisms of the carbohydrate metabolism of this important pathogen.

It is the loss of function of PP2Acα holoenzymes that results in the accumulation of amylopectin in T. gondii tachyzoites. Although it inhibits the activity of both PP1 and PP2A, OA has different potencies on them in mammalian cells (7, 17, 39). Specifically, it has been reported that OA at 50 nM does not inhibit the recombinant PP1 of T. gondii (18). However, we have shown here, at this same concentration, that it results in amylopectin granules in the tachyzoites, suggesting that OA inhibits PP2A. However, little is known about PP2A, including the holoenzyme components and its function in the glycogen metabolism in T. gondii and other apicomplexans (40, 41), although limited information is available in yeast and mammals (42). There are four catalytic subunits of the PP2A subfamily in T. gondii: PP2Acα (phenotype score: −1.54), PP2Acβ (phenotype score: −4.39), PP4c (phenotype score: −4.79), and PP6c (phenotype score: −4.05) (19, 43–45). We have tried to knock out each one of them in the current study. Unfortunately, only PP2Acα was successfully knocked out, which fortunately resulted in a phenotype of amylopectin accumulation in the tachyzoites of T. gondii. Failure in the generation of knockouts of the rest of catalytic subunits of the PP2A subfamily suggests that they are more crucial than PP2Acα for the survival of the parasite. Different from the fact that the complete loss of PP2Acα results in early embryonic lethality at stage E6.5 in mice (46, 47), the ensured knockout of PP2Acα does not kill T. gondii, which should be due to a compensatory effect of PP2Acβ, PP4c, or PP6c, and this enables us to explore the roles of this phosphatase in the carbohydrate metabolism of this parasite in detail.

A PP2A holoenzyme heterotrimer that is composed of PP2Acα, the scaffolding subunit A (TGGT1_315670), and the regulatory subunit B′/PR61 is involved in the amylopectin metabolism of T. gondii. It is known that a PP2A core enzyme usually exists in the form of a heterodimer, consisting of one catalytic subunit and one scaffolding subunit, and interacts with different regulatory subunits to regulate a wide range of biological processes (28, 48, 49). Having identified that PP2Acα is one of the catalytic subunits that is required for the metabolism of amylopectin in T. gondii, we elected to identify the regulatory subunit that is involved in this holoenzyme, as only one A subunit has been reported in this parasite (19, 50). In mammals, the binding of the PP2A core enzyme (PP2Acα/A) to a B subunit is regulated by reversible methylation, involving two methylation regulators, namely, LCMT1 and PME1 (31). However, although these proteins are predicted to exist in T. gondii (19), they did not play a role in the metabolism of amylopectin, based on our results. Indeed, with functional screening, we found that only PR61 was unaffected by the methylation regulators. AlphaFold (51, 52) enabled the high-accuracy structural modeling of PP2Acα, the scaffolding subunit A, and the B′/PR61 of T. gondii as well as the visualization of the PP2A heterotrimer assembled by these subunits *in silico*, indicating a functional holoenzyme heterotrimer. Although elevated S6K phosphorylation and reduced glycogen accumulation have been reported in PR61 knockout flies (49), multiple experiments in the current work consistently support the claim that a deficiency of B′/PR61 is involved in the amylopectin accumulation in the tachyzoites of T. gondii. Therefore, B′/PR61 might play a distinct role between apicomplexans and other species (e.g., yeast and flies) (42, 49, 53), which warrants further investigation.

A deficiency of the PP2Acα-B′/PR61 holoenzyme affected the glucose metabolism and energy homeostasis in tachyzoites as well as the subsequent intracellular growth and virulence of T. gondii. Although both glucose and glutamine are important carbon sources for energy metabolism in T. gondii (25, 26), we demonstrated that glucose is the carbon source for the polysaccharide granules that were observed in the Δ*PP2Acα* knockout. Based on the results of decreased ATP production and marked metabolomic changes in aspects of amylopectin, glycolysis, the TCA cycle, and the PPP in Δ*PP2Acα*, we hypothesized that the PP2Acα-B′/PR61 holoenzyme is required for glycolysis or the pentose phosphate pathway in T. gondii and its disruption leads to carbon flow to the polysaccharide synthesis pathway, which causes the accumulation of amylopectin and further affects the metabolism of amino acids, fatty acids, etc., essentially due to insufficient energy production in the fast-replicating tachyzoites. Due to the metabolic disorder in T. gondii, the physiological processes, such as the invasion of tachyzoites into host cells and intracellular replication, cannot be driven by enough energy, which significantly inhibits the *in vitro* proliferation process. Our findings in the type I strain of T. gondii are somewhat supported by the results of a recent study in the type II strains (ME49 and Pru) of this parasite, in which knocking out PP2Acα resulted in the excessive accumulation of starch and interrupted tachyzoite-bradyzoite differentiation (54). An amylopectin metabolism disorder might affect the transformation of bradyzoites to tachyzoites as well as the infectivity or viability of T. gondii and related apicomplexans (cf. 54–59). Indeed, significantly inhibited intracellular proliferation and decreased virulence during acute infection in mice were observed in Δ*PP2Ac*α tachyzoites, strongly demonstrating the importance of the PP2Acα-B′/PR61 holoenzyme in the viability of T. gondii. More information on the roles of amylopectin metabolism in bradyzoites, coupling the transcriptional changes of the marker genes *bag1*, *eno1*, or *ldh2*, should provide insights into the roles of PP2Acα in the chronic infection stage of T. gondii. Although only select metabolites (including fatty acids and carbohydrates) of metabolism pathways of interest were analyzed in the current study, abundance alterations of other metabolites, such as organic acids and derivatives, organic oxygen compounds, and organoheterocyclic compounds were also detected in Δ*PP2Acα*. A better understanding of these metabolomic changes should underpin the carbohydrate biology in this and related parasites.

Studies have shown that *Tg*PP2A can regulate the metabolism of amylopectin by regulating Ca^2+^-dependent protein kinase (CDPK2) phosphorylation (54). However, in mammals, PP2A has been found to have extensive regulatory effects on the insulin signaling pathway and mitogen-activated protein kinase (MAPK) signaling pathway (50, 60). Therefore, a comprehensive investigation of the molecules that are involved in these pathways will contribute to a better understanding of the PP2Acα-associated carbon metabolism in T. gondii. The current study has raised many more important questions that need to be answered. (i) The substrates of PP2A in regulating the metabolism of T. gondii need to be elucidated, especially whether there is a unique regulatory mechanism from its mammalian hosts. (ii) Further investigations are needed to elucidate the mechanisms by which PP2A governs spatially complex and temporally regulated molecular events that occur during tachyzoite-to-bradyzoite differentiation. (iii) The potential of the PP2Acα-B′/PR61 holoenzyme as an intervention target or vaccine antigen is also unknown.

In conclusion, we have identified a PP2Acα-B′/PR61 holoenzyme that is involved in glucose metabolism in the tachyzoites of T. gondii, and its deficiency suppresses the proliferation and virulence of this important zoonotic parasite. This pivotal discovery lays a solid foundation for a novel strategy for the intervention of *Toxoplasma* infection and toxoplasmosis.

## MATERIALS AND METHODS

### Ethics statement.

The use of the experimental animals in this study was approved by the Experimental Animal Ethics Committee of Zhejiang University (approval no. ZJU201308-1-10-072). The handling of animals strictly followed the Guidelines for the Use of Experimental Animals of the People's Republic of China.

### Parasites, cell lines, antibodies, and animals.

RHΔ*ku80* tachyzoites of T. gondii were maintained in culture as described previously (60). Transgenic T. gondii was constructed from parental cells as described previously (61, 62). The HFF-1 and HEK 293T cells were purchased from the Cell Bank of the Chinese Academy of Sciences (Beijing, China). The anti-SAG1, inner membrane complex (IMC1), Toxoplasma gondii (*Tg*), PP2Acα, LCMT1, and PME1 polyclonal antibodies were prepared in the lab as described previously (63). The goat anti-rabbit (or mouse) IgG (H+L) highly cross-adsorbed secondary antibody and Alexa Fluor Plus 594 (or 488/647) were obtained from Invitrogen (Shanghai, China). HA-Tag (C29F4) rabbit MAb was purchased from CST (MA, USA). The New Zealand rabbits and ICR female mice were obtained from the SLAC Laboratory Animal Co., Ltd. (Shanghai, China).

### Preparation of polyclonal antibodies.

The coding sequences of *TgPP2Acα*, *TgLCMT1*, and *TgPME1* were obtained via PCR amplification, individually inserted into pET30a, and verified via sequencing. The recombinant proteins were expressed in E. coli BL21 (SunYa, China) and purified using Ni-Agarose Resins (Sangon Biotech, China). The recombinant protein was mixed with Freund's adjuvant. Mice and rabbits were first immunized using 100 μg and 200 μg of protein, respectively, with second and third immunizations being performed on days 14 and 28. The serum was obtained 7 days after the last boost.

### Western blot (WB).

Cells or tachyzoites were lysed in a radio-immunoprecipitation assay (RIPA) lysis buffer (Beyotime Biotechnology, China) supplemented with a protease inhibitor cocktail (Bimake, USA) at 4°C for 1 h. The whole cellular lysate was collected from the supernatants via centrifugation at 12,000 × *g* at 4°C for 10 min. The soluble proteins were separated via sodium dodecyl sulfate-polyacrylamide gel electrophoresis (SDS-PAGE) and then transferred to a polyvinylidene fluoride (PVDF) membrane (Millipore, USA). The membrane was probed with the primary antibody (1:1,000), incubated with the HRP-conjugated secondary antibody (1:5,000), and exposed to electrochemiluminescence (ECL) substrates (Fude, China). Signals were captured with the exposure meter ChemiDoc chemiluminescence system (Bio-Rad, USA).

### Coimmunoprecipitation.

Coimmunoprecipitation (Co-IP) was performed as described previously (64). LCMT1-HA, PME-1-HA, or the parental vector plasmid was cotransfected with PP2Acα-FLAG into HEK 293T, respectively. Cells were harvested 24 h after transfection and lysed in RIPA as the input *in vitro*. LCMT1-HA or PME1-HA endogenously labeled tachyzoites were lysed in RIPA as the input in T. gondii. Cellular lysates of the HEK 293T cells were incubated with anti-Flag affinity gel (Bimake, USA) overnight at 4°C. After the affinity gel was fully washed, anti-FLAG/HA/tubulin antibodies were used in Western blot analyses. T. gondii tachyzoites (LCMT1-HA, PME1-HA, or PR61-HA) lysates were incubated overnight at 4°C with protein A/G magnetic beads (Bio-Make, China) that had previously been incubated with PP2Acα polyclonal antibodies at room temperature for 30 min. Anti-PP2Acα/HA/SAG1 antibodies were used in Western blotting. HA-Tag (C29F4) Rabbit MAb, (Cell Signaling Technology, USA), FLAG-Tag (D6W5B) Rabbit MAb (Cell Signaling Technology, USA), and FD β-tubulin (Fude, China) were used at a dilution of 1:2,000. The primary antibodies, namely, mouse anti-SAG1 (1:1,000) and rabbit anti-PP2Acα (1:1,000), were prepared and preserved in the lab.

### Immunofluorescence assay.

An immunofluorescence assay (IFA) was performed as described previously (65). In brief, *Toxoplasma* tachyzoites of different treatments were cocultured with HFF-1 cells on coverslips in a 24-well plate in high glucose DMEM (BI, Israel) supplemented with 20% fetal bovine serum (FBS) (Gibco, South America) and 100 U/mL penicillin plus 100 μg/mL streptomycin (Gibco, USA). The medium was regularly changed as needed. The cells on coverslips were washed twice in phosphate-buffered saline (PBS, pH = 7.4) and fixed with 4% paraformaldehyde (PFA) at room temperature for 15 min. This was followed by permeabilization in 0.2% Triton X-100 at 37°C for 15 min. The permeabilized cells were blocked with 1% bovine serum albumin (BSA) in PBS at 37°C for 1 h and incubated with primary antibodies. Then, the cells were incubated with Alexa Fluor-conjugated secondary antibody (Invitrogen, USA) at 37°C for 1 h. 4′,6-diamidino-2′-phenylindole (DAPI; Sigma, USA) was used to stain the nucleus at room temperature for 5 min. The coverslips were mounted and observed under an LSM880 confocal microscope (Carl Zeiss, Germany). Rabbit monoclonal anti-HA Tag (C29F4) antibodies (Cell Signaling Technology, USA) were used at a dilution of 1:1,000. The primary antibodies, namely, mouse anti-*Tg* (1:1,000), mouse anti-IMC1 (1:1,000), mouse anti-LCMT1 (1:1,000), mouse anti-PME1 (1:1,000), rabbit anti-IMC1 (1:1,000), and rabbit anti-PP2Acα (1:1,000) were prepared and preserved in the lab.

### Plaque assay.

Freshly harvested tachyzoites (*n* = 200) were used to infect HFF-1 cells cultured in high glucose DMEM (BI, Israel) containing 20% fetal bovine serum (FBS) (Gibco, South America) in a 6-well plate. After 7 days of culture, the cells on coverslips were fixed with methanol for 30 min and then stained with 0.2% crystal violet in PBS for 30 min, and this was followed by two washes in PBS and air drying (23). The numbers and areas of plaques were counted/measured using ImageJ (National Institutes of Health, USA) and analyzed using the GraphPad Prism 8 software (GraphPad Software, USA). Three independent replicates were performed for each treated tachyzoite, including the WT, OA-treated tachyzoites, Δ*PP2Acα*, Δ*LCMT1*, Δ*PME1*, and Δ*PR61*. In the plaque assay for the OA treated group, the extracellular tachyzoites were collected and incubated with 0 nM, 50 nM, or 2 μM OA for 3 h before infecting HFF-1 cells cultured with a normal culture medium.

### Adhesion and invasion assays.

HFF-1 cells in 24-well plates were infected with the WT, OA-treated extracellular tachyzoites, Δ*PP2Acα*, Δ*LCMT1*, or Δ*PME* tachyzoites (5 × 10^6^ tachyzoites/well) for 20 min at 37°C. HFF-1 monolayers were washed three times with PBS (pH = 7.4) and then fixed with 4% PFA at room temperature for 15 min. Extracellular parasites were labeled with mouse anti-*Tg* antibodies and goat anti-mouse IgG Alexa Fluor 594. Then, the monolayers were permeabilized with 0.25% Triton X-100 and blocked with 1% BSA, and this was followed by the labeling of all of the intracellular and extracellular parasites with rabbit anti-IMC1 antibodies and goat anti-rabbit IgG Alexa Fluor 488. The nuclei were stained with DAPI (63). At least 10 images for each sample were taken stochastically to count the numbers of parasites and host nuclei under an LSM880 confocal microscope. The adhesion efficiency was measured by the average number of attached and invaded (labeled with Alexa Fluor 488) tachyzoites per host cell. The invasion efficiency was measured by the average number of invaded (only labeled with Alexa Fluor 488 but not Alexa Fluor 594) tachyzoites per host cell. The relative invasion efficiency was calculated as a mean percentage of invaded parasites to the total number of parasites. In the adhesion and invasion assays for the OA-treated group, the tachyzoites were collected and incubated with 0 nM, 50 nM, or 2 μM OA for 1 h before infecting HFF-1 cells cultured with a specific concentration of OA.

### Intracellular replication assay.

*Toxoplasma* tachyzoites (WT, OA-treated tachyzoites, Δ*PP2Acα*, Δ*LCMT1*, or Δ*PME1*) were added into HFF-1 cells (10^5^ tachyzoites/well on a coverslip) in a 24-well plate. After being incubated at 37°C for 3 h, the cells on coverslips were washed with PBS (pH = 7.4) and then cultured in fresh, high-glucose DMEM supplemented with 20% FBS and 100 U/mL penicillin plus 100 μg/mL streptomycin. The cells on coverslips were fixed with 4% PFA at room temperature for 15 min, and this was followed by permeabilization in 0.2% Triton X-100 at 37°C for 15 min, and then blocking with 1% BSA in PBS (pH = 7.4) at 37°C for 1 h. The permeabilized intracellular parasites were labeled with rabbit anti-IMC1 antibodies and goat anti-mouse IgG Alexa Fluor 594. At least 50 parasitophorous vacuoles (PV) were used to calculate the number of (labeled with Alexa Fluor 594) tachyzoites per PV as described previously (63). In the intracellular replication assay for the OA treated group, the tachyzoites-infected HFF-1 cells were cultured in the culture medium with 0 nM, 50 nM, or 2 μM OA.

### Transmission electron microscopy (TEM).

Freshly collected T. gondii tachyzoites were fixed in 2.5% glutaraldehyde overnight and then in 1% osmium solution for 2 h. To observe the ultrastructure of the OA-treated group, the tachyzoite was incubated with OA for 3 h before fixation. The fixed tachyzoites was dehydrated for 15 min each in 30%, 50%, 70%, and 80% ethanol, and this was followed by 90% and 95% acetone solutions, and, finally, by pure acetone twice for 20 min each time. The dehydrated samples were treated with a mixture of the Spurr embedding agent and acetone at a 1:1 or 3:1 ratio for 1 h or 3 h, respectively. Then, the samples were transferred to a pure embedding agent resin at room temperature overnight. After osmotic treatment, the samples were embedded, heated at 70°C overnight, and sliced using a LEICA EM UC7 ultrathin slicer (Leica Microsystems, Germany). The sliced sections were stained in lead citrate solution for 5 min and uranyl acetate solution for 10 min. The ultrastructure of T. gondii tachyzoites was visualized under a Hitachi H-7650 transmission electron microscope (Hitachi, Japan).

### Polysaccharide staining.

The polysaccharides of the WT, OA-treated tachyzoites, Δ*PP2Acα*, Δ*LCMT1*, Δ*PME1*, or Δ*PR61* cells (*n* = 5 × 10^5^) (14, 66) were detected with a Glycogen Periodic Acid-Schiff (PAS) Stain Kit (For Cells) (Solarbio, China). In brief, freshly harvested T. gondii tachyzoites were added into 24-well plates with coverslips of confluent HFF-1 monolayers for 24 h or 60 h, and they were then fixed in 4% PFA overnight. The fixed tachyzoites were incubated with periodic acid at room temperature for 20 min. After extensive washing, the samples were reacted with Schiff’s reagent for 20 min and were then observed under an Olympus CX21 microscope (Olympus Life Science, Japan). In the PAS assays for the OA-treated groups, the extracellular tachyzoites were collected and incubated with 0 nM, 50 nM, or 2 μM OA for 3 h before being fixed.

### Quantification of glycogen.

Glycogen in the tachyzoites of T. gondii was quantified using a Glycogen Content Detection Kit (Solarbio, China), according to the manufacturer’s instructions. After filtration and centrifugation, the tachyzoites (*n* = 5 × 10^8^) egressing from the HFF-1 cells were broken apart via ultrasonication for 30 cycles of 3 s and 10 sec intervals at W = 20%. Glycogen was extracted using a strong alkaline extraction solution from the kit in a boiling bath, incubated with an anthrone chromogenic agent under strongly acidic conditions in the boiling bath (67), and determined using a multiscan spectrum (Thermo Fisher Scientific, USA).

### Assay for PP2A activity.

The activity of PP2A in *Toxoplasma* tachyzoites was detected using a PP2A Activity Colorimetric Assay Kit (GENMED, China). After 10^9^ freshly harvested tachyzoites (WT, Δ*PP2Acα*, and ::*PP2Acα*) were cleaned with reagent A from the kit, reagent B was added to decompose the tachyzoites. Then, the instructions of the kit were followed, and the samples were put into a multiscan spectrum (Thermo Fisher Scientific, USA) to determine the free phosphates that were released after the reaction with the specific phosphorylated polypeptide PKpTIRR. The PP2A phosphatase activity was calculated according to the standard curve.

### Metabolomics assay.

The tachyzoites of RHΔ*ku80* and Δ*PP2Acα* were added into HFF-1 cells *in vitro* for 2 to 3 days. The freshly egressed parasites were collected and filtered using a 5 μm filter. To extract metabolites from tachyzoites, chilled methanol:acetonitrile (1:1, vol/vol) was added to each sample and vortexed at 4°C to remove the protein and extract the metabolites. After the centrifugation, the supernatant was collected and dried in a vacuum centrifuge. The samples were redissolved in 100 μL acetonitrile/water (1:1, vol/vol) solvent for an LC-MS analysis, which was performed using a UHPLC (1290 Infinity LC, Agilent Technologies, USA) that was coupled to a quadrupole time-of-flight (AB TripleTOF 6600, AB SCIEX, USA) at Shanghai Applied Protein Technology (APT, Shanghai).

For the separation of the metabolites, each sample was analyzed using a 2.1 mm × 100 mm ACQUIY UPLC BEH 1.7 μm column (Waters, Ireland) with a flow rate of 0.5 mL/min at 25°C. The mobile phase consisted of solvent A (25 mM ammonium acetate and 25 mM ammonium hydroxide in water) and solvent B (acetonitrile). The following gradient was applied: 95% B for 0 to 0.5 min; a linear reduction to 65% for 0.5 to 7 min, a linear reduction to 40% for 7 to 8 min, kept for 1 min; and an increase to 85% in 6 s, employing a 3 min re-equilibration period. The eluted metabolites were further analyzed in both negative and positive electrospray ionization modes with the IonSpray Voltage Floating ± 5500 V. In MS-only acquisition, the TOF mass range was set from 60 to 1,000 Da, and the scan time was 0.2 s. In auto MS/MS acquisition, all precursors were fragmented using 35 ± 15 eV, and the range was set from 25 to 1,000 Da. The scan time was set to 0.05 s.

The following parameters were used for peak picking: centWave *m/z* = 25 ppm, peakwidth = c (10, 60), prefilter = c (10, 100), and, for peak grouping: bw = 5, mzwid = 0.025, and minfrac = 0.5. The identification of metabolites was performed by comparing the accurate *m/z* value (<25 ppm) and MS/MS spectra with an in-house database that was established with the available authentic standards. After sum-normalization, the processed data were subjected to a multivariate data analysis. Sevenfold cross-validation and response permutation testing were used to evaluate the robustness of the model. The variable importance in the projection (VIP) value of each variable in the OPLS-DA model was calculated to indicate its contribution to the classification. A Student’s *t* test was applied to determine the significance of differences between the RHΔ*ku80* and Δ*PP2Acα* of independent samples. A VIP value of >1 and a *P* value of <0.05 were used to screen significantly changed metabolites. A Pearson’s correlation analysis was performed to determine the correlation between two variables. All of the metabolites that were detected in positive and negative ion modes were analyzed based on a univariate analysis, and the metabolites with a fold change (FC) of >1.5 or a FC of <0.67 and a *P* value of <0.05 were visualized via volcano plots.

### Quantitative real-time PCR (qRT-PCR).

Gene transcription in the T. gondii tachyzoites was determined using qRT-PCR as described previously (63). Total RNAs were extracted from tachyzoites using the TRIzol reagent (Invitrogen, USA). Complementary DNA (cDNA) was synthesized via the reverse transcription of 1 μg of total RNA using a ReverTra Ace qPCR RT Kit (TOYOBO, Japan). Diluted cDNA was used to amplify targeted sequences using a ChamQ SYBR qPCR Master Mix Kit (High ROX Premixed; Vazyme, China) and suitable primers, as listed in Table S1, in a CFX96 Touch detection system (Bio-Rad, USA). β-tubulin was used as an internal control for a relative expression/fold change analysis using the 2^−ΔΔCT^ method (68). Three independent replicates were included for each analysis.

### Virulence test.

Six-week-old female ICR mice (SLAC, China) were intraperitoneally injected with 100 freshly isolated tachyzoites per mouse. 10 mice per group were infected with the WT, Δ*PP2Acα*, ::*PP2Acα*, or Δ*PR61 Toxoplasma* tachyzoites. The mice were raised under well-controlled conditions and monitored daily for their survival for 30 days or until death. Serum was collected from the surviving mice, and the T. gondii infections were determined via ELISA. An OD ratio P/N of >2.1 was considered positive for a *Toxoplasma* infection.

### Quantification and statistical analysis.

Statistical analyses were performed using GraphPad Prism 8 (GraphPad Software, USA). The data were presented as the mean ± standard deviation (SD). A Student’s *t* test or a two-way analysis of variance (ANOVA) was used to assess the significance of differences between or among groups. A *P* value of ≤0.05 was considered to be indicative of a statistically significant result.

### Data availability.

All data generated or used in this study are presented in the paper and the supplemental material.
