# Supplementary material for: PP2Acα-B′/PR61 Holoenzyme of Toxoplasma gondii Is Required for the Amylopectin Metabolism and Proliferation of Tachyzoites
Source: Microbiol Spectr. 2023 May 18;11(3):e00104-23. doi: 10.1128/spectrum.00104-23 (PMC10269777; doi:10.1128/spectrum.00104-23)
Supplement: Supplemental file 1 — Fig. S1 to S4 and Table S1. Download spectrum.00104-23-s0001.pdf, PDF file, 1.3 MB [file spectrum.00104-23-s0001.pdf]

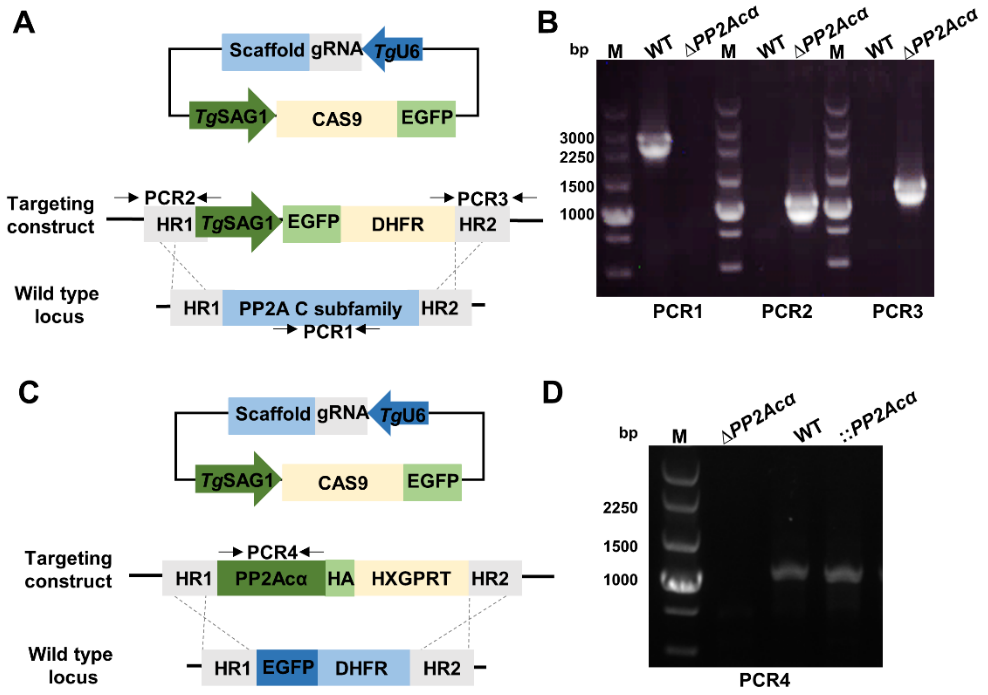

E

Group	Positive control	1	2	3	4	5	6	7	8	9	10	Negative control
P/N	9.23	9.19	9.17	9.24	9.24	9.24	9.24	9.24	9.24	9.23	9.23	0.75

Fig S1. Knockout and add-back of *PP2Acα* gene in tachyzoites of *Toxoplasma gondii*. (A) Schematic illustration of knocking out PP2Ac subfamily by CRISPR/Cas9-mediated homologous gene replacement in the RHΔ*ku80*Δ*hxgprt* cells (RHΔ*ku80*). (B) Electrophoresis of PCR amplicons as indicated WT: Parental cells. (C) Schematic illustration of adding back *PP2Acα* by CRISPR/Cas9-mediated homologous gene replacement in the Δ*PP2Acα* knockouts. (D) Electrophoresis of PCR amplicons as indicated. WT: Parental cells. (E) Positive/negative (P/N) OD ratios of antibodies in the serum of mice survived from Δ*PP2Acα* tachyzoites infection. P/N ratio greater than 2.1 is considered positive for *Toxoplasma* infection.

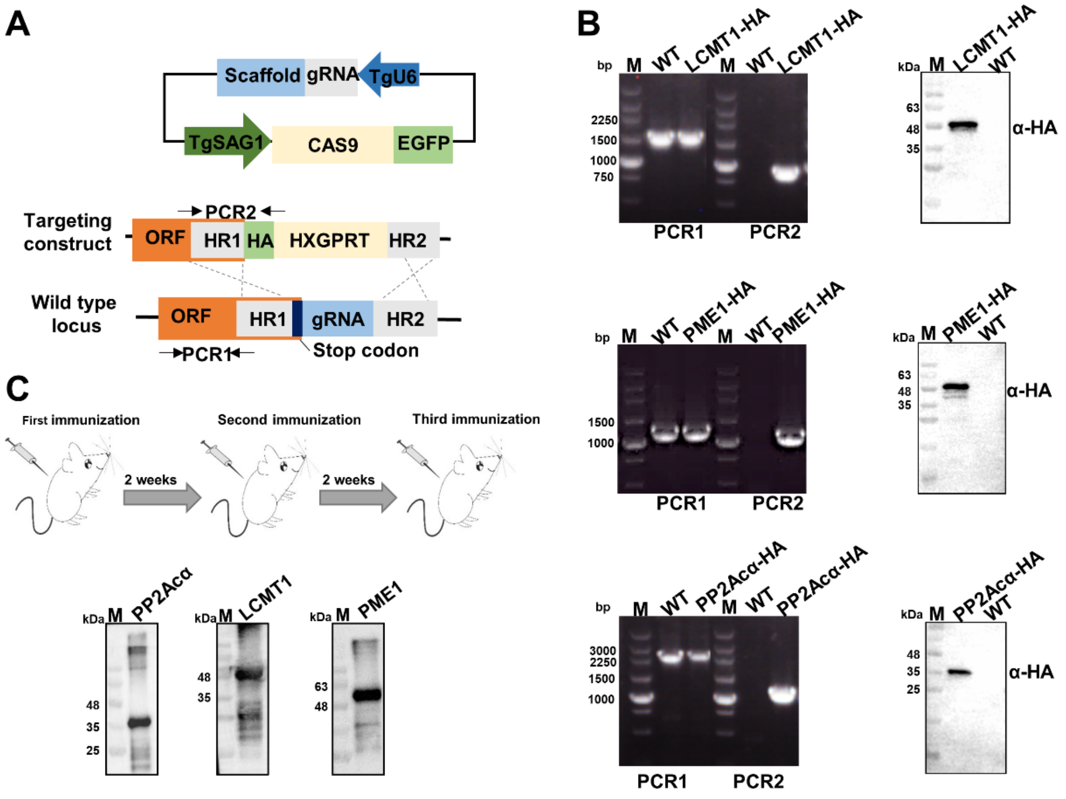

Fig S2. Endogenous 3× hemagglutinin tagged cells and polyclonal antibodies against PP2Acα and its methylation regulators. (A) Schematic illustration of PP2Acα-3HA, leucine carboxyl methyl transferase 1 (LCMT1) -3HA and protein phosphatase methylesterase 1 (PME1)-3HA by CRISPR/Cas9 tagging strategy in RHΔ*ku80*. Three consecutive hemagglutinin (HA) tags are included. (B) PCR (left) and Western blot (WB, right) of representatives expressing PP2Acα-HA, LCMT1-3HA and PME1-3HA. Anti-HA antibodies are used in Western blot. (C) Western blotting analysis of polyclonal antibodies against PP2Acα, LCMT1 or PME1.

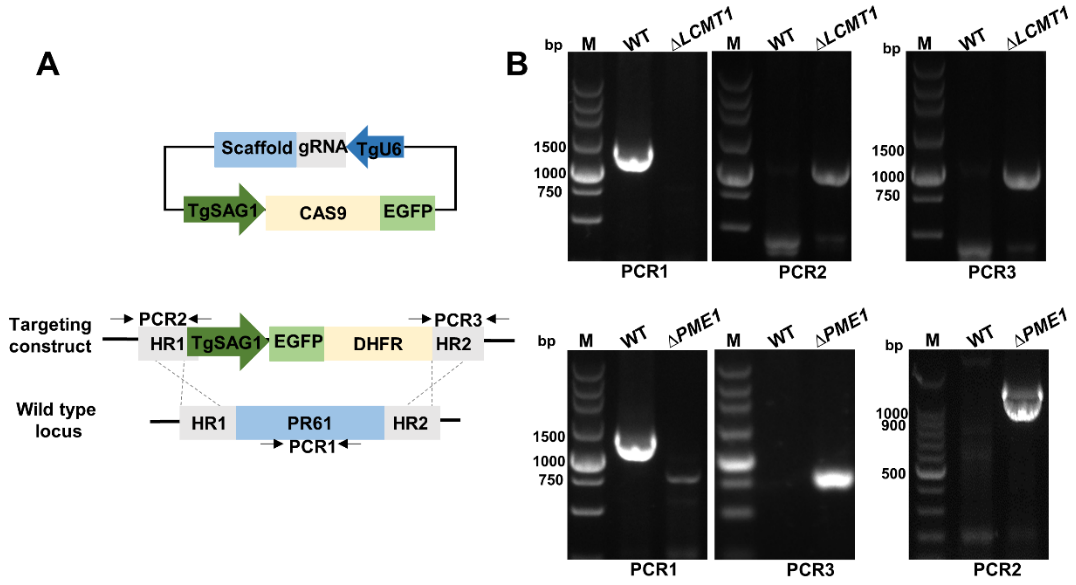

Fig S3. Knockout of *LCMT1* and *PME1* genes in tachyzoites of *Toxoplasma gondii*. (A) Schematic illustration of the genetic deletion of *LCMT1* and *PME1* by CRISPR/Cas9 tagging strategy in RHΔ*ku80*. WT: parental cells; Δ*LCMT1*: tachyzoite with LCMT1 knockout; Δ*PME1*: tachyzoite with PME1 knockout. (B) PCR identification of the Δ*LCMT1* or Δ*PME1* representative clones.

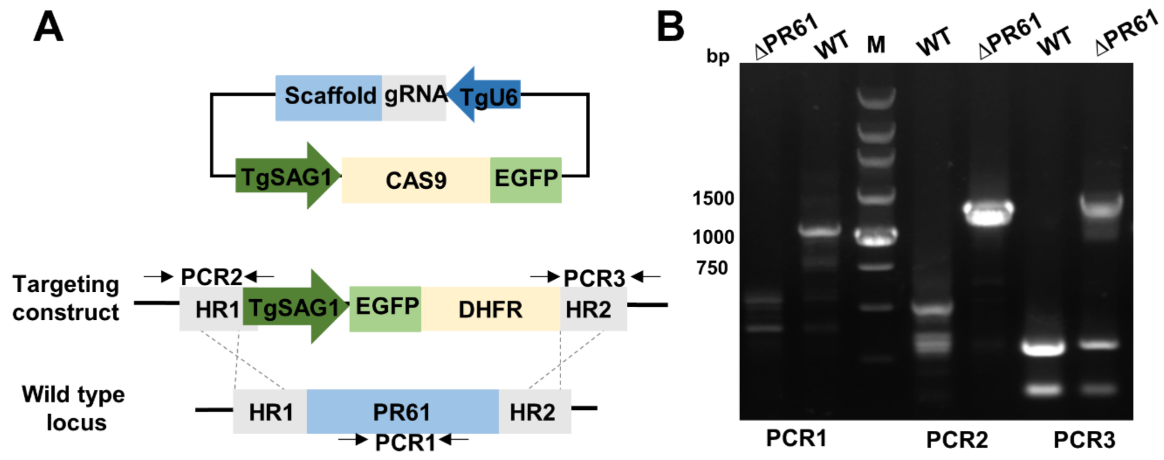

Fig S4. Knockout of B'/*PR61* in tachyzoites of *Toxoplasma gondii*. (A) Schematic illustration of knocking out B'/PR61 by CRISPR/Cas9-mediated homologous gene replacement in the RHΔ*ku80*. WT: parental cells; Δ*PR61*: tachyzoite with PR61 knockout; (B) PCR identification of the Δ*PR61* tachyzoites representative clones.

Table S1 Primers used in the current study.

Name	Sequence (5' - 3')
PP2Acα-KO sgRNA F	AGTTGTTGGGGCAAGAAGAGTTGCG
PP2Acα-KO sgRNA R	AAACCGCAACTCTTCTTGCCCCAAC
PP2Acα-KO donor F	TTTTTCTTCCACACACTCTGCCGTTTCTCCTTTCTTGTCACCTCCGCAAAGCTTTTACATCCGTTGCCTTTTC
PP2Acα-KO donor R	CGTTTGTCCTGGCCTCCTCAAGCCTTCGTTTGTTTTGTCGGTCTCCCCGGGGGCAAGAATTGTGTTAACCGGT
PP2Acα-KO screen F1	GAGTGCCTGCGCAAATATGG
PP2Acα-KO screen R1	GCAGAAGCGACGGATTGAAC
PP2Acα-KO screen F2	TCAGATCCCTGGAGACATCCGAA
PP2Acα-KO screen R2	GTTGCGGCACTGTGAGCACTGAT
PP2Acα-KO screen F3	GCTTCCTATTCGCTTTTGACGCT
PP2Acα-KO screen R3	TTCTGTTCACCGACTTTTTTTGC
PP2Acα-Comp sgRNA F	AGTTACACGCTTTGTGGTGAGAAG
PP2Acα-Comp sgRNA R	AAACCTTCTCACCACAAAGCGTGT
PP2Acα-Comp donor F	TTTTTCTTCCACACACTCTGCCGTTTCTCCTTTCTTGTCACCTCCGCAATGTCGGGTGCAACTCCA
PP2Acα-Comp donor R	TCTTTTCTTCGCATTCTTCCTCATCGCCTCGCATCCTTCTCGCCTGTGATAGGGCGAATTGGAGCTCC
PP2Acα-Comp screen F1	GAGTGCCTGCGCAAATATGG
PP2Acα-Comp screen R1	GCAGAAGCGACGGATTGAAC
PP2Acβ-KO sgRNA F	AGTTAGAGGCGCCGGGATGCTCTG
PP2Acβ-KO sgRNA R	AAACCAGAGCATCCCGGCGCCTCT
PP2Acβ-KO donor F	GCTGCACAAGTTGAGCACTCCCGTTCTTGACGCGAGTAGCTGTACCAAGCTTTTACATCCGTTGCCTTTTCCA
PP2Acβ-KO donor R	GTTGAACAGCAAGCGGTCGAGCGAAGAAGACTGCAGCCTAGTCATCTCGGGGGCAAGAATTGTGTTAACCGGTTC
PP4c-KO sgRNA F	AGTTATGGGAGCAGCACCGTTTGG
PP4c-KO sgRNA R	AAACCCAAACGGTGCTGCTCCCAT
PP4c-KO donor F	CCTCCTCCTCACTCTTTCCGTCTACGCGTTCTGCTGCTCTCTCCTCTCAAGCTTTTACATCCGTTGCCTTTTCCA
PP4c-KO donor R	AGAGACGCGGAATTCGAAGAGAACGTTGAACTCCAAACGTCGCCTGCTGGGGGCAAGAATTGTGTTAACCGGTTC

PP6c-KO sgRNA F	AGTTGCGACTGGCAAGACTTGGTCA
PP6c-KO sgRNA R	AAACTGACCAAGTCTTGCCAGTCGC
PP6c-KO donor F	GCATCGCTTCGTTCTCCCACAGACCAGCACTGGCGGGTCCGACACCAGAAGCTTTTACATCCGTTGCCTTTTCCA
PP6c-KO donor R	TCCCCCTGCCTACCTCCTCCTCCCCTCCCCCTACGCATACGCTGTATCGGGGGCAAGAATTGTGTTAACCGGTTC
LCMT1-KO sgRNA F	AGTTGTCAGGCTCACTACTCGATTG
LCMT1-KO sgRNA R	AAACCAATCGAGTAGTGAGCCTGAC
LCMT1-KO donor F	GAGTCGCGTGTCTCAGTGATCAGTCACGCCCGAGCACACGAGATAATTAAGCTTTTACATCCGTTGCCTTTTC
LCMT1-KO donor R	GAGGTGCCACGAGGACAAAGAATTTGAGTGTGGAACCTAGTGGACTTCGGGGGCAAGAATTGTGTTAACCGGT
LCMT1-KO screen F1	TGAAATCCGCTCCCAGTGTC
LCMT1-KO screen R1	GGGTCATCTTCTCGTTGCCA
LCMT1-KO screen F2	GGACGGTTTGAAGGCGTTTC
LCMT1-KO screen R2	CACGCGTCTCAAGGTTTGTG
LCMT1-KO screen F3	TGCAGGTGTCACATCCACAA
LCMT1-KO screen R3	CTCCTAGGTGTCTCGTTGGC
PME1-KO sgRNA F	AGTTAAAGTGGGCGGAGGCTCCCG
PME1-KO sgRNA R	AAACCGGGAGCCTCCGCCCACTTT
PME1-KO donor F	GTCGCTTGCTCCTCGAGTGTCAAGCCGGCCTCGCCTCTCGTCGAAAAGAAGCTTTTACATCCGTTGCCTTTTC
PME1-KO donor R	CGTGGGGTCGTCGACTTCAGGCGGCTTCCTAGTGTATGCACACTGCGTGGGGGCAAGAATTGTGTTAACCGGT
PME1-KO screen F1	AAGAGGCTTCCAACGGTGTT
PME1-KO screen R1	CCCTGCACAGATAAGCACCT
PME1-KO screen F2	AGGACTTTATGCGGCCTTCC
PME1-KO screen R2	CTACGAAACGTTCACACGCC
PME1-KO screen F3	TTCACCGCCGAGGATTTTGA
PME1-KO screen R3	GCAACGAAACAGACAACGCA
PR61-KO sgRNA F	AGTTGCCTCCGCCAGGAGGAGGCG
PR61-KO sgRNA R	AAACCGCCTCCTCCTGGCGGAGGC

PR61-KO donor F	GGTCTGCCTTGCGTCTCAGTTTGCTCCCATAATGCTTTCGACTCTCTCCCGGTAGCCTCCAAGCTTTTACATCCGTTGCCTTTTC
PR61-KO donor R	GTCAGAAACTTTGAGGAAGACGTCGAACGCGCACCACCTTCTCCCGCGAAGAGCGGAGACGGGGGCAAGAATTGTGTTAACCGGT
PR61-KO screen F1	ATTCTGCTGAACACGCAACCTC
PR61-KO screen R1	AAAAGTCGCGTCGGCGGA
PR61-KO screen F2	CGGCATTTTCCATTCGGCA
PR61-KO screen R2	TCAAATGCCTCGTGTGGGAA
PR61-KO screen F3	TCAAATGCCTCGTGTGGGAA
PR61-KO screen R3	GCTGCGCCTTCTGTCAAAAA
PP2Acα-HA sgRNA F	AGTTGAACCACGGCGCGTTTGTCC
PP2Acα-HA sgRNA R	AAACGGACAAACGCGCCGTGGTTC
PP2Acα-HA donor F	CAAGCCTCGGGAGCAGACATGCAGAAACGAATTCCGGACTACTTCCTGGGCGGAGAAATCTCCCTGAGAA
PP2Acα-HA donor R	TCTTTTCTTCGCATTCTTCCTCATCGCCTCGCATCCTTCTCGCCTGTGATAGGGCGAATTGGAGCTCC
PP2Acα-HA screen F	GCCGAACTATTGCTACCGCTGT
PP2Acα-HA screen R	GCCATCGGACATAGGACTCATT
LCMT1-HA sgRNA F	AGTTGGACTCCAATCAGTTAGCCG
LCMT1-HA sgRNA R	AAACCGGCTAACTGATTGGAGTCC
LCMT1-HA donor F	GAGAAGATGACCCTGCCGCGCCTGAAGCAGATCTACGAAGGCATGAATGGCGGAGAAATCTCCCTGAGAA
LCMT1-HA donor R	AAAAGCGCGGCTTCCGAGGAGTCGGAGGAAAGAATCTCAATTCACAGAATAGGGCGAATTGGAGCTCC
LCMT1-HA screen F	GTGGGGCAGAGTTCTCAAAG
LCMT1-HA screen R	CCATCGGACATAGGACTCAT
PME1-HA sgRNA F	AGTTGTGGGGTCGTCGACTTCAGG
PME1-HA sgRNA R	AAACCCTGAAGTCGACGACCCCAC
PME1-HA donor F	TCTGTCCAGCCTTGGCGCGTTCACCACTCTGCGCCGAAGCCTGCGAGTGGCGGAGAAATCTCCCTGAGAA
PME1-HA donor R	CATGTACAGACATTACCAAACTGAGGATTTACATGTGGGGACAGGTGAATAGGGCGAATTGGAGCTCC
PME1-HA screen F	CTGGACCTGGCGAACAGATT
PME1-HA screen R	CTCCTATCGGAGAGGGGGAG

PR61-HA sgRNA F	AGTTGTACACATATACGTGAGGAG
PR61-HA sgRNA R	AAACCTCCTCACGTATATGTGTAC
PR61-HA donor F	GGCGGGGAGGACGCGTTCTTGGTTCGCTTTTCCGCCGACGCGACTTTTGGCGGAGAAATCTCCCTGAGAA
PR61-HA donor R	AACTTTGAGGAAGACGTCGAACGCGCACCACCTTCTCCCGCGAAGAGCATAGGGCGAATTGGAGCTCC
PR61-HA screen F	ACCAGCAACTCACCTACTGC
PR61-HA screen R	CCCCCGGACAGAACAATACC
PP2Acα qPCR F	GTCACCATTTTCTCTGCGCC
PP2Acα qPCR R	CTTGCAGAAGCGACGGATTG
PP2Acβ qPCR F	ATTGGGTCCGAGTGCTTCTG
PP2Acβ qPCR R	TGTCGTAGGGGTCTGACCAT
PP4c qPCR F	GAGCTCTTTCGTGTGGGGAA
PP4c qPCR R	GGGGTACCGAACTTTCAGGG
PP6c qPCR F	GGGGCCTCACATGGAATCAA
PP6c qPCR R	ACGCCTTCTTGTCGAGAGTG
PP1c qPCR F	CACGGATTGCTTCAACTGCC
PP1c qPCR R	CAGTGGGCCTGACAATCCTT
PR53 qPCR F	TTCTTCCCCGCTCTTTCGAC
PR53 qPCR R	GGAAACGCATGAAAGCCTGG
PR61 qPCR F	GTGGAACAACGAGCACATCG
PR61 qPCR R	CCTCTTGCTGCTTGCGTTTT
PR70 qPCR F	CCACTACCTCTGGTCTCGGA
PR70 qPCR R	CCAAGCACCGTGGTTTTCAG
LCMT1-HA F	TAGAAGACACCGACTCTAGAATGTCGTCTTATTTATCGTC
LCMT1-HA R	TTGTAATCCAGAGGTTGATTTCAAGCGTAGTCTGGGACGTCGTATGGGTAATTCATGCCTTCGTAGATCT
PME1-HA F	TAGAAGACACCGACTCTAGAATGGATAACGACTTCTTTCTTCC
PME1-HA R	TTGTAATCCAGAGGTTGATTTCAAGCGTAGTCTGGGACGTCGTATGGGTAACTCGCAGGCTTCGGCGCA

PP2Acα-Flag F	TAGAAGACACCGACTCTAGAATGTCGGGTGCAACTCCA
PP2Acα-Flag R	TTGTAATCCAGAGGTTGATTTTACTTATCGTCATCGTCTTTGTAATCCAGGAAGTAGTCCGGAATTC
LCMT1-pET30a F	CGGGATCCATGTCGTCTTATTTATCGTCTCCCC
LCMT1-pET30a R	GCTCTAGAATTCATGCCTTCGTAGATCTGCTT
PME1-pET30a F	CGGAATTCATGGATAACGACTTCTTTCTTCCAC
PME1-pET30a R	CCGCTCGAGACTCGCAGGCTTCGGCGCAGAGTG
PP2Acα-pET30a F	CGGGATCCATGTCGGGTGCAACTCCAG
PP2Acα-pET30a R	GCTCTAGATCACAGGAAGTAGTCCGGAATTCGT
